# Crosstalk between codon optimality and *cis*-regulatory elements dictates mRNA stability

**DOI:** 10.1186/s13059-020-02251-5

**Published:** 2021-01-05

**Authors:** Santiago Gerardo Medina-Muñoz, Gopal Kushawah, Luciana Andrea Castellano, Michay Diez, Michelle Lynn DeVore, María José Blanco Salazar, Ariel Alejandro Bazzini

**Affiliations:** 1 Stowers Institute for Medical Research, 1000 E 50th St, Kansas City, MO 64110 USA; 2 Present Address: National Laboratory of Genomics for Biodiversity (LANGEBIO), Unit of Advanced Genomics, 36824 Irapuato, Mexico; 3 Present Address: Instituto de Fisiología Celular, Universidad Nacional Autónoma de México, 04510 México City, Mexico; 4 Department of Molecular and Integrative Physiology, University of Kansas Medical Center, 3901 Rainbow Blvd, Kansas City, KS 66160 USA

**Keywords:** Codon optimality, MicroRNA, miR-430, m6A, Zebrafish, *Xenopus*

## Abstract

**Background:**

The regulation of messenger RNA (mRNA) stability has a profound impact on gene expression dynamics during embryogenesis. For example, in animals, maternally deposited mRNAs are degraded after fertilization to enable new developmental trajectories. Regulatory sequences in 3′ untranslated regions (3′UTRs) have long been considered the central determinants of mRNA stability. However, recent work indicates that the coding sequence also possesses regulatory information. Specifically, translation *in cis* impacts mRNA stability in a codon-dependent manner. However, the strength of this mechanism during embryogenesis, as well as its relationship with other known regulatory elements, such as microRNA, remains unclear.

**Results:**

Here, we show that codon composition is a major predictor of mRNA stability in the early embryo. We show that this mechanism works in combination with other *cis*-regulatory elements to dictate mRNA stability in zebrafish and *Xenopus* embryos as well as in mouse and human cells. Furthermore, we show that microRNA targeting efficacy can be affected by substantial enrichment of optimal (stabilizing) or non-optimal (destabilizing) codons. Lastly, we find that one microRNA, miR-430, antagonizes the stabilizing effect of optimal codons during early embryogenesis in zebrafish.

**Conclusions:**

By integrating the contributions of different regulatory mechanisms, our work provides a framework for understanding how combinatorial control of mRNA stability shapes the gene expression landscape.

## Background

A large body of work has identified 3′ untranslated regions (3′UTRs) as possessing mRNA stability determinants [1, 2]. For example, microRNAs, mRNA modifications, and some RNA binding proteins affect mRNA stability by recognizing regulatory elements mainly in the 3′UTR [3–7] or across the entire mRNA [8–13]. These *cis*-regulatory elements can, in turn, affect how a cell grows, differentiates, and responds to its environment [2, 14]. Regulation of mRNA stability plays a critical role during the maternal-to-zygotic transition (MZT). In all animals, after fertilization, maternally deposited transcripts are degraded while the zygotic genome is activated [15, 16]. For example, a zygotically expressed microRNA family, miR-430/-427/-309, is responsible for the repression of a subset of maternal mRNAs in zebrafish, *Xenopus*, and *Drosophila*, respectively [15, 17–20]. Other factors contribute to the clearance of maternal mRNAs, such as the reader protein YTHDF2 that binds and destabilizes N6-methyladenosine (m6A)-modified mRNAs in vertebrates [4, 10, 21]. Despite the identification of these and other factors, it is still unclear how most maternal mRNAs are degraded [15].

Recent studies have shown that translation affects mRNA stability in a codon-dependent manner, and codon content influences the clearance of maternal mRNAs during the MZT [22, 23] and during homeostasis in human cell lines [24]. This mechanism, called codon optimality, refers to the ability of a particular codon to affect the stability of an mRNA *in cis* [25]. Therefore, codons that enhance mRNA stability have been defined as “optimal” and codons that decrease mRNA stability as “non-optimal.” This codon-mediated regulation is present from bacteria to vertebrates [22–26]. Quantitative modeling has revealed that codon content is the primary determinant of mRNA stability in yeast [27]. However, in vertebrates, the strength of this mechanism, as well as its relationship with other known regulatory elements (e.g., microRNAs, m6A), remains unclear.

Here, we show that codon content predicts mRNA stability during both early embryogenesis and homeostasis. In the presence of active *cis*-regulatory elements, microRNAs and mRNA methylation, we find that the codon effect is still observed. However, microRNAs can reduce codon-mediated stabilization and codon content can impact microRNA targeting efficacy. Together, these results indicate that mRNA stability in vertebrates is dictated through the combined activities of the coding and 3′UTR sequences. In sum, our results provide the foundation for considering how the entire mRNA sequence affects stability in *cis*, rather than continuing to focus solely on distinct *cis*-regulatory elements within the 3′UTR or in the coding sequence.

## Results

### Genome-wide prediction of mRNA stability based on codon content in vertebrates

**Fig. 1:**
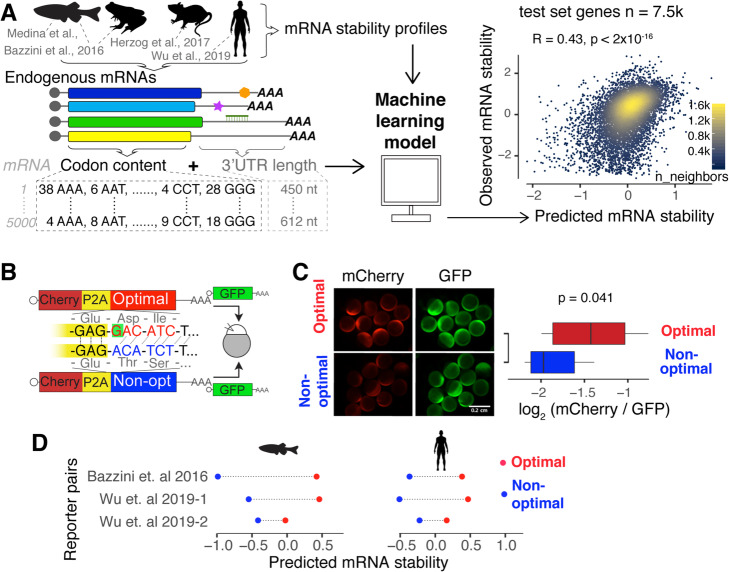
Codon composition predicts mRNA stability in vertebrates. **a** Scheme of the procedure to train a predictive model of mRNA stability. For each endogenous mRNA, the codon frequencies and the 3′UTR length are used as predictors to train a lasso regression model [28]. The scatter plot shows the point density of predicted and observed mRNA stability (test set genes *n* = 7576, Pearson correlation test). **b** Scheme of the 1 nucleotide out of frame reporters (optimal and non-optimal): two mRNAs that differ in the codon composition due to a single nucleotide deletion (G in red, highlight with green) which creates a frameshift. The encoding mCherry fluorescent protein was followed by a *cis*-acting hydrolase element (P2A) and then by a coding region enriched in optimal or non-optimal codons due to the frameshift. P2A causes ribosome skipping; therefore, the mCherry is not fused to the optimal or non-optimal encoded proteins. The mRNA reporter pairs were co-injected with mRNA encoding for GFP as an internal control [24]. **c** Fluorescence microscopy images of representative embryos at 8 h post-injection (hpi) with the indicated 1 nt out of frame reporter and GFP. Box plot displays fluorescence quantification at 8 hpi with each reporter. The mCherry fluorescence intensity was normalized to GFP intensity in each embryo (*p* = 0.041, paired *t* test). **d** mRNA stability predictions for 1 nucleotide out of frame reporters in fish and human cells. In all cases, the prediction for the optimal reporter is higher than that for the non-optimal (*p* = 0.007, binomial test)

Translation strongly affects mRNA stability *in cis* in a codon-dependent manner [22–25]. Therefore, we hypothesized that codon content can predict mRNA stability in vertebrates. To test this hypothesis, we trained a machine learning model [28] that predicts mRNA stability as a function of only codon frequency and transcript length (Fig. 1a; see the “Methods” section). The model was trained to account for endogenous mRNA stability profiles that have either been previously published and/or generated after blocking transcription in zebrafish and *Xenopus* embryos [22] (Fig. 1a; Additional file 1: Fig. S1; Additional files 2, 3, 4: Table S1–3), human cell lines [24], and mouse embryonic stem cells [29]. We observed that the position of the codon along the transcript can affect the codon-mediated effect on mRNA stability [23, 30] (Additional file 1: Fig. S2a); however, the model does not gain significant predictive power by including codon positional information (Additional file 1: Fig. S2b). Our model, which for example, ignores *cis*-regulatory elements in the 3′UTR, explains 19% (*R*^2^ = [0.170, 0.202] bootstrap 95% CI, test data) of the variation in mRNA degradation rates across vertebrates (Fig. 1a and Additional file 1: Fig. S3a-c and S4).

To verify that the model predicts mRNA stability based on codon content rather than the primary nucleotide sequence, we tested whether our model could predict observed differences in the expression of reporter mRNAs that contain similar nucleotide sequences but different codon content due to an engineered frameshift [22, 24, 31]. These frameshift reporters uncouple nucleotide sequence differences from codon differences due to the conversion of the reading frame from being enriched in optimal codons “optimal reporter” to non-optimal codons “non-optimal reporter” by a single nucleotide insertion in the middle of a nearly 1500-nucleotide sequence. We have previously shown that these reporter mRNAs display differential expression in zebrafish embryos and human cells [22, 24] (Fig. 1b). For example, a pair of coding sequences where mCherry follows a ribosome skipping sequence (P2A) and a coding region that is enriched in either optimal or non-optimal codons (due to a one nucleotide frameshift) were injected into one-cell stage zebrafish embryos. At 8 h post-injection (hpi), embryos injected with the optimal reporter displayed a higher level of mCherry fluorescence than embryos injected with the non-optimal reporter (Fig. 1c). Interestingly, for three pairs of frameshifted reporters [22, 24] (Fig. 1c), the model correctly predicted the optimal reporter mRNA to be more stable than the non-optimal reporter mRNA in zebrafish and humans (Fig. 1d), despite sharing almost identical nucleotide sequences. This result indicates that our model predicts mRNA stability based on codon content, rather than nucleotide composition.

### Codon optimality is the predominant determinant of mRNA stability during the maternal-to-zygotic transition

**Fig. 2:**
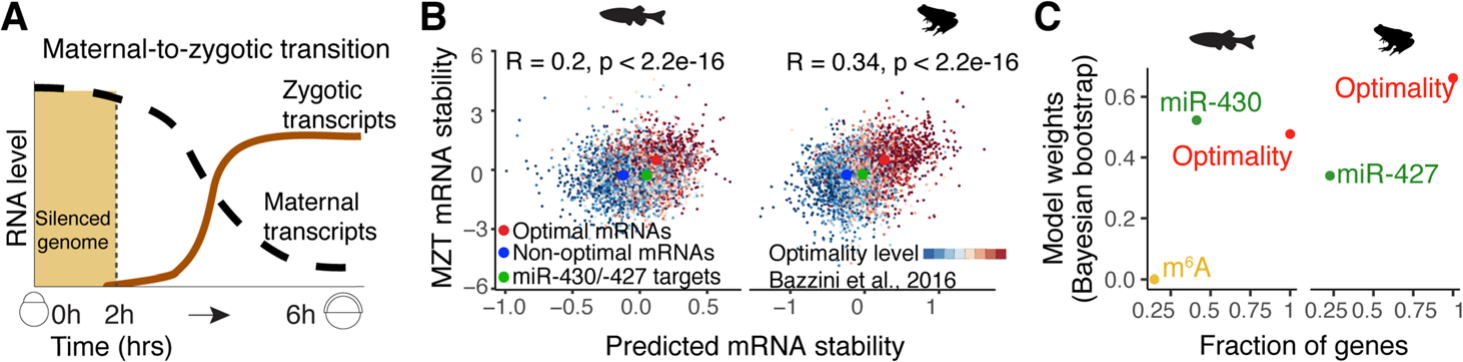
Codon optimality is the major determinant of mRNA stability. **a** Diagram depicting the maternal to zygotic transition in zebrafish. **b** Scatter plots of predicted and observed mRNA stability, during MZT, for maternal mRNAs in zebrafish and *Xenopus*. The gradient of color represents the content of optimal codons [22]. The predicted mRNA stability correlates with the proportion of optimal codons (*p* < 2 × 10^−16^, Pearson correlation). The mRNA stability median of mRNAs enriched in optimal (red) or non-optimal (blue) codons, as well as mRNAs with miR-430/-427 target sites in the 3′UTR (green) are shown. **c** Codon content explains most of the mRNA decay during MZT. The *x*-axis shows the fraction of coding genes that can be regulated by different mRNA stability pathways. For microRNAs, this fraction corresponds to the number of seed sites (GCACTT) in the 3′UTR, and for m6A, the fraction is the number of target genes reported [10]. The *y*-axis shows the Bayesian model comparison weights [32]. These weights represent which model is more likely to predict unobserved data better, higher values indicate stronger regulatory effects

During the MZT, maternal mRNAs are degraded and zygotic gene expression is activated (Fig. 2a) [5]. Our model was trained with mRNA decay profiles in the absence of zygotic transcription by transcriptional inhibition with alpha-amanitin. We first evaluated whether our model could still predict mRNA stability during MZT for maternal mRNAs when zygotically derived decay programs are active (for example miR-430/-427 in zebrafish and *Xenopus*, respectively). We defined mRNA stability during the MZT as the log_2_-fold change in mRNA levels between 2 and 6 h post-fertilization (hpf) for zebrafish (Additional file 1: Fig. S1a-b and Additional 2: Table S1) and 2 and 9 hpf for *Xenopus* [33]. By not taking into account the repressive activity of microRNAs, we found that our model reliably overestimated the stability of mRNAs that contain miR-430 or miR-427 target sites (within 3′UTRs) in zebrafish and *Xenopus*, respectively (Fig. 2b). Nonetheless, we found a significant correlation between the predicted mRNA stability based on codon composition and the observed stability during zebrafish and *Xenopus* MZT (*R* = 0.21 and 0.34, *p* < 2 × 10^−16^, zebrafish and *Xenopus*, respectively) (Fig. 2b). Similar results were obtained when using multiple timepoints across the MZT in zebrafish (Additional file 1: Fig. S3d and Additional file 2: Table S1) and *Xenopus* [33], highlighting the prominent and dynamic role of this gene regulation program (Additional file 1: Fig. S3d). Together, these results indicate that regulation via codon optimality is globally evident, even in the presence of another widespread regulatory mechanisms (e.g., miR-430/-427).

Next, we interrogated how the regulatory strength of codon optimality compares to other known regulatory programs during the MZT, such as microRNAs and RNA methylation (m6A). We defined microRNA regulation for endogenous genes as the number of 6-mer “seed” sequences (GCACTT, miR-430 in zebrafish and miR-427 in *Xenopus*) present in 3′UTRs. For m6A regulation, the published proposed targets were used [10]. Finally, we employed the log_2_-fold change expression values (zebrafish = 6 hpf/2 hpf, *Xenopus* = 9 hpf/2 hpf) for maternally provided mRNAs in zebrafish (Additional file 1: Fig. S1a-c and Additional file 2: Table S1) and *Xenopus* [33], as an indicator of mRNA stability during MZT. Interestingly, a Bayesian model comparison analysis [32, 34] shows that codon optimality is the most likely program to best estimate mRNA stability in both species (Fig. 2c). Both m6A and microRNA pathways also partly explain mRNA behavior but only a fraction of mRNAs are regulated by these pathways (Fig. 2c). Together, these results suggest that codon optimality is the most pervasive determinant of mRNA stability during early embryogenesis.

### The unexplained mRNA decay by codon composition highlights *cis*-regulatory elements

**Fig. 3:**
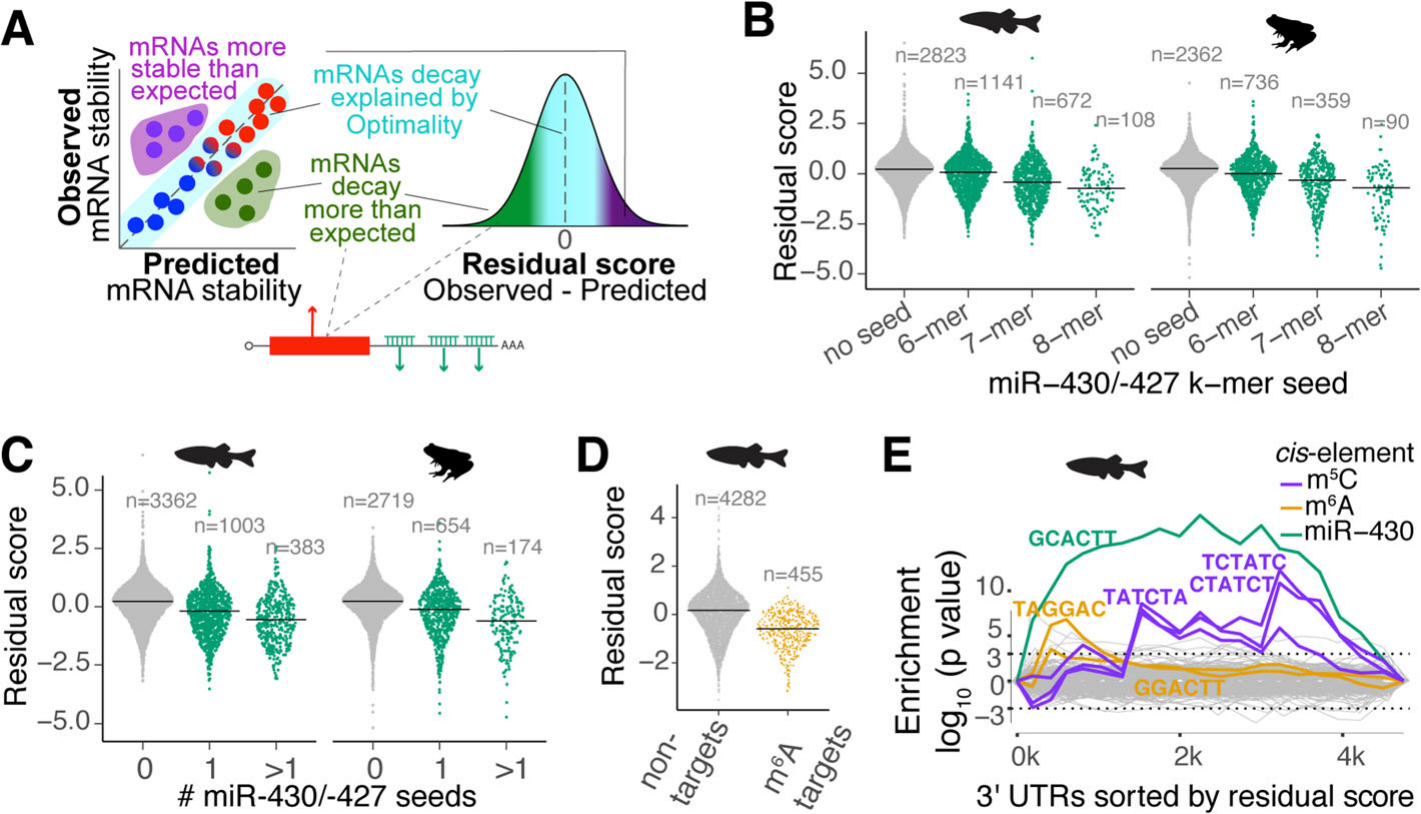
Dissecting *cis*-regulatory elements after accounting for codon-mediated regulation. **a** Diagram describing the residual score (observed − predicted). mRNAs that decay more than expected by the model show negative residuals and potentially contain destabilizing *cis*-regulatory elements. The mRNAs with positive residual scores might have stabilizing *cis*-regulatory elements. **b**, **c** The model overestimates the mRNA stability of miR-430 targets. Sinaplot showing the distribution of the residual scores for targets and not targets of miR-430. In this type of plot, each dot represents an individual mRNA. The targets of miR-430 are grouped by the type of miR-430 seed (**b**) (*p* < 2 × 10^−16^, ANOVA test) or by the number of target seeds (**c**) (*p* < 2 × 10^−16^, ANOVA test) present in the 3′UTR during the MZT in zebrafish (Additional file 1: Fig. S1a-b) and *Xenopus* [33]. **d** The model also overestimates the stability of mRNAs that contain m6A methylation mark. Sinaplot showing that m6A targets [10] display lower residual score distribution than not targets during the MZT in zebrafish (*p* < 2 × 10^−16^, *t* test). **e** Sylamer landscape plot that tracks occurrence biases of 6-nucleotide words in the 3′UTRs using hypergeometric *p* values for all words across the mRNAs ranking based on residual values [35]. The highlighted 6-mers are significantly associated with mRNA stabilization (TATCTA, CTATCT, and TCTATC) and destabilization (GCACTT, TAGGAC, and GGACTT). The color shows the putative regulatory pathway that recognizes these 6-mers. The dotted line shows a hypergeometric *p* = 0.01

Following our results showing that codon optimality is the most pervasive determinant of mRNA stability during MZT, and given that miR-430/-427 target stability was overestimated by our model (which does not account for microRNA activity) (Fig. 2), we hypothesized that mRNA decay behavior that cannot be explained by codon optimality likely stems from the presence of other *cis-*regulatory elements, potentially in the 3′UTR (e.g., miR-430/-427) (Fig. 3a; Additional file 1: Fig. S5a). To explore this idea, we further analyzed the unexplained model variation by assessing the difference between observed mRNA stability during MZT and predicted mRNA stability (i.e., residual scores; Fig. 3a; Additional file 1: Fig. S5a and Additional file 5: Table S4). We observed that miR-430/-427 target genes displayed negative residual scores during MZT in zebrafish and *Xenopus* (Fig. 3b; Additional file 1: Fig. S5a), and these scores correlated with the number of microRNA target sites in the 3′UTR, as well as predicted microRNA target site strength (Fig. 3b, c) [18, 20]. Previously defined m6A target mRNAs also displayed negative residual scores (Fig. 3d), consistent with repressive activity previously attributed to the m6A modification [10]. Furthermore, maternal mRNAs with the m6A motif in the coding region exhibited lower residual scores when compared to mRNAs possessing the m6A motif in the 3′UTR during MZT in both zebrafish and *Xenopus* embryos (Additional file 1: Fig. S5e). Together, these results indicate that our model can account for the repressive activity of microRNAs and m6A RNA methylation, opening the possibility for detecting regulation by other *cis*-regulatory elements.

To further understand the unexplained mRNA decay by codon composition, we analyzed the residual scores with Sylamer [35]. Sylamer is a method for detecting enriched *k*-mer signals from a ranked gene list. When genes were ranked by the residual score (Table S4), miR-430/-427 [18] and m6A- [10] associated *k*-mers in the 3′UTR were enriched in destabilized mRNAs at multiple time points across the MZT (Fig. 3e; Additional file 1: Fig. S5d). Moreover, a set of 6-mers resembling the binding site for Ybx1, which recognizes m^5^C-modified mRNAs, were enriched in the 3′UTR of stable mRNAs, consistent with m^5^C stabilizing activity in zebrafish [36] (Fig. 3e). As expected, none of these *k*-mer elements was detected when ranked according to predicted stability based on our model (which only accounts for codon-mediated effects) (Additional file 1: Fig. S5b). Interestingly, *k*-mer enrichment scores for these elements were less significant when mRNAs were sorted according to log_2_-fold change during the MZT (rather than by residual score; Additional file 1: Fig. S5c), suggesting that codon optimality obscures the regulatory effects of these *cis*-elements. Hence, analyzing the variation in mRNA degradation after accounting for codon optimality is a novel approach to reveal the regulatory effects of other regulatory programs.

### Codon optimality, microRNAs, and m6A act in conjunction to regulate gene expression in vertebrates

**Fig. 4:**
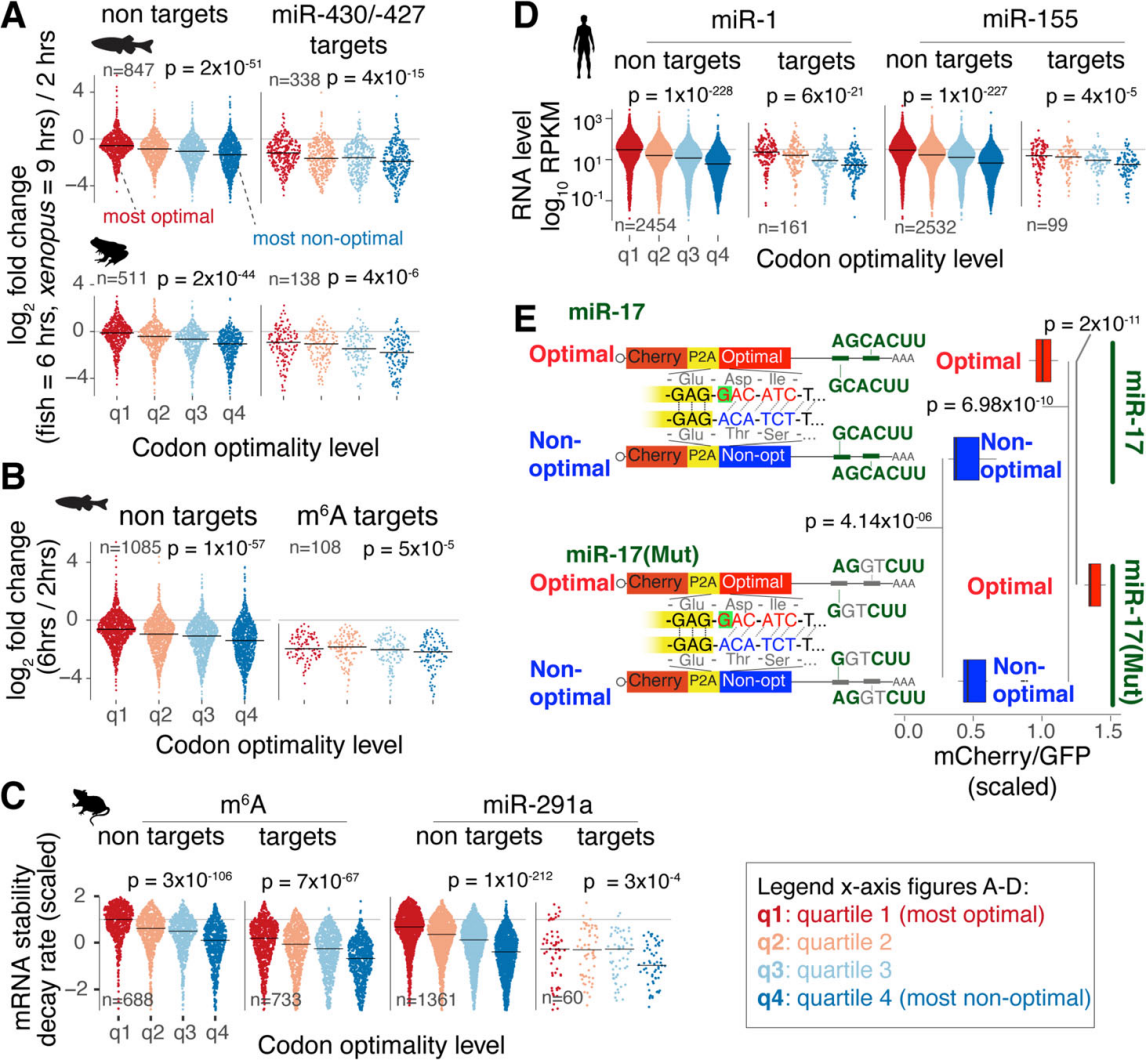
Codon optimality affects mRNA stability and gene expression of microRNA targets and m6A targets in vertebrates. mRNAs were divided into targets (microRNA or m6A) and nontargets. Each group was divided into four equal groups with decreasing levels of optimal codons. All *p* values were computed with a linear model. **a** Sinaplot showing the distribution of mRNA stability during MZT (log_2_-fold change 6 h post-fertilization (hpf)/2 hpf) for targets and not target genes of miR-430/-427 in zebrafish (Additional file 1: Fig. S1a-b) and *Xenopus* [33] embryos. **b** Sinaplot showing the distribution of mRNA stability during MZT in zebrafish for methylated (m6A) and non-methylated mRNAs [10]. **c** Sinaplot showing the mRNA stability distribution of m6A and miR-291a targets [29] and not targets in mouse embryonic stem cells. **d** Sinaplot showing the RNA-level distribution of miR-1 and miR-155 targets [37] after microRNA transfection in human cells. **e** Diagram depicting reporter genes containing almost identical nucleotide sequence but different codon composition (enriched in optimal or non-optimal codons) due to a single insertion changing the frame. For each coding region, a 3′UTR sequence containing miR-17 seed sites (7-mer AGCACTT) or a mutant version with two nucleotides mutated disrupting the miR-17 seed sites were cloned. The boxplot shows the distribution of scaled mCherry/GFP intensity for reporters with and without miR-17 seed site in transfected 293T human cells. Both coding sequence and miR-17 affect the reporter expression (*p* values computed with paired *t* test)

Most of the research in post-transcriptional gene regulation has focused solely on a single regulatory program at a time [10, 18, 22] but has not yet established how multiple programs operate in conjunction to define mRNA stability. We hypothesized that regulatory pathways operate in combination either additively or antagonistically, to generate a variety of mRNA stability patterns. To test this hypothesis, we investigated whether the codon optimality effect on mRNA stability can be detected in mRNAs that are regulated by *cis*-elements (e.g., microRNAs and/or m6A) during the zebrafish (Table S1; Additional file 1: Fig. S1a-c) and *Xenopus* [33] MZT. First, two groups of maternal mRNAs for each species were created: the miR-430/-427 target and non-target groups [18, 19]. The target group contains mRNAs with at least one miR-430/-427 seed site (6-mer GCACUU) in the 3′UTR (zebrafish *n* = 1380, *Xenopus n* = 588). The non-target groups do not contain miR-430/-427 seed sites (zebrafish *n* = 3357, *Xenopus n* = 2007). Next, within each group, mRNAs were divided into quartiles based on their codon composition (most optimal to least optimal) (Fig. 4a, Additional file 1: Fig. S4, and Additional file 6: Table S5). As expected, the miR430/-427 target groups were less stable than the control group in both species (*p* < 2 × 10^−9^, paired *t* test) (Fig. 4a). Interestingly, in both groups, increased codon optimality was associated with increased mRNA stability (*p* < 4 × 10^−6^, regression analysis). Similar results were observed across multiple time points during the zebrafish and *Xenopus* MZT (Additional file 1: Fig. S6a). Moreover, comparable outcomes were observed for a subset of mRNAs that were experimentally validated as under miR-430 regulation [18] (*R* = 0.24, *p* = 0.0017, regression analysis) (Additional file 1: Fig. S6b), suggesting that the stabilizing effects of codon optimality remain evident for transcripts under active demonstrated microRNA regulation [18]. Similarly, we found that increased codon optimality is also associated with increased mRNA stability for transcripts reported as m6A targets [10] during the zebrafish MZT (Fig. 4b).

We next assessed how codon optimality influences miR-291 and m6A regulation in mouse embryonic stem cells. The m6A and miR-291 target transcripts display decreased stability compared to control groups (*p* < 2 × 10^−5^ linear regression) (Fig. 4c) [4, 38]. However, as seen in zebrafish and *Xenopus*, in mouse, m6A and miR-291 target transcripts enriched in optimal codons were more stable than targets containing fewer optimal codons (Fig. 4c). Similarly, while transfection of miR-1 or miR-155 into human cells [37] affects mRNA levels of their respective targets (*p* < 2 × 10^−10^, one-tailed Kolmogorov–Smirnov (K–S) test), both miR-1 and miR-155 targets enriched in optimal codons displayed higher mRNA levels than targets enriched in non-optimal codons (*p* < 3 × 10^−4^, linear regression) (Fig. 4d). These results suggest that endogenous gene expression profiles are shaped by the additive effects of codon content and additional *cis*-regulatory elements.

The contribution of codon optimality and microRNAs to dictate the level of expression was also observed for fluorescence reporters containing optimal or non-optimal coding sequences paired with active or inactive microRNA target sites (Fig. 4e; Additional file 7: Table S6) [18]. Upon transfection into human 293T cells, higher fluorescence intensities were observed for both optimal reporters (with or without microRNA target sites) when compared to non-optimal reporters (with or without microRNA target sites) (*p* < 6.98 × 10^−10^, paired *t* test), highlighting that for these reporters the influence of codon optimality is stronger than miR-17-mediated repression (Fig. 4e). Furthermore, we observed significantly decreased fluorescence intensity from the optimal reporter when paired with the microRNA target site (*p* = 2.10 × 10^−11^, paired *t* test), highlighting the ability of miR-17 to reduce the mRNA expression of its counterpart reporter enriched in optimal codons (Fig. 4e). However, those differences due to the microRNA were lower for the non-optimal reporters (*p* = 4.14 × 10^−06^, paired *t* test) (Fig. 4e). In sum, all these results (Fig. 4) suggest that both coding sequence and elements in the 3′UTR work in combination to regulate mRNA expression.

### Targeting efficacy of miR-430/-427 can be affected by the coding sequence

**Fig. 5:**
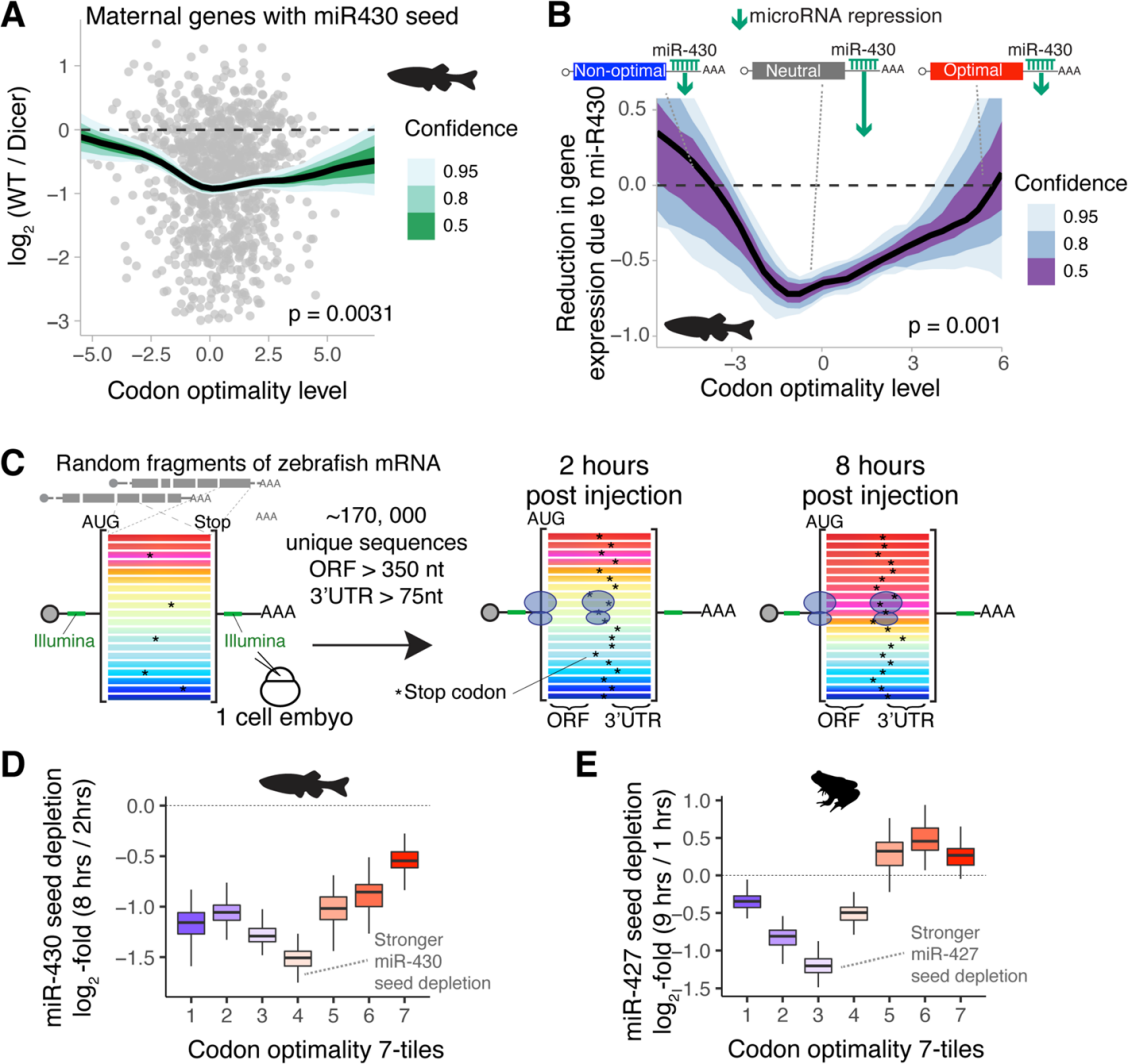
Targeting efficacy of miR-430/-427 can be affected by the coding sequence. **a** Scatter plot comparing codon optimality level and change in expression between wildtype zebrafish embryos vs maternal/zygotic Dicer mutant embryos at 6 h post-fertilization [39]. Only mRNAs with miR-430 seed in the 3′UTR (GCACTT) are shown. The line represents the average change in expression (log_2_-fold WT/Dicer at 6 hpf) as a function of the codon optimality level (Additional file 1: Table S5). The effect of miR-430 is not constant across different levels of codon optimality (*p* value was obtained using an *F* test comparing a model with a non-linear effect on codon optimality vs constant effect). The confidence interval was determined with bootstrap replicates (*n* = 100) [40]. **b** Line plot showing the expected decrease in gene expression due to miR-430 during zebrafish MZT (log_2_-fold change 6 vs 2 hpf) (Additional file 1: Fig. S1a-b). The *y*-axis represents a measure, estimated from the data, of the miR-430 repressive strength with respect to codon optimality (*x*-axis). For example, for an mRNA that is very repressed by miR-430, the miR-430 component will be larger (higher negative value in the *y*-axis). However, for another gene, which has a weak miR-430, the miR-430 component is smaller (closer to 0 in the *y*-axis). The *p* value denotes the statistical significance of the non-linear interaction between codon optimality and miR-430 presence (*F* test) obtained with a generalized additive model [41]. The confidence interval was determined with bootstrap replicates (*n* = 100) [40]. **c** Scheme of the reporter library which includes random fragments of the zebrafish transcriptome [22]. Transcripts share the same 5′ and 3′UTR but some sequences contain a stop codon in the coding region. These stop codons create a random and longer 3′UTR sequence. mRNAs were injected at the one‐cell stage in zebrafish [22], and the reporter library is analyzed at 2 and 8 hpi using high-throughput sequencing. To analyze the depletion of miR-430 with respect to the codon content of the transcripts, we filtered those sequences that contain a coding region of at least 350 nucleotides and a random 3′UTR length of at least 75 nucleotides. **d**, **e** Analysis of miR-430/427 depletion in the reporter library. The reporters were grouped into 7-tiles, equal size, with increasing levels of codon optimality (Additional file 1: Fig. S5c). For each tile, we computed the depletion of the miR-430/427 seed (GCACTT) in the 3′UTR (log_2_-fold 8 h/2 h for zebrafish and 9h/1 h for *Xenopus*). Each boxplot is formed by bootstrap replicates (*n* = 100) [40]

We next asked how extreme optimal or non-optimal codon composition affects microRNA repression. Four sets of evidence indicate that microRNA efficacy is diminished for highly optimal and non-optimal target mRNAs. First, we observed that the most miR-430 responsive targets during MZT are not enriched in optimal or non-optimal codons but contain average codon optimality (Fig. 5a; Additional file 6: Table S5). Specifically, the miR-430 targeting efficacy was based on the log_2_-fold change of mRNAs with a miR-430 target site (6-mer) in the 3′UTR between wildtype and MZ*dicer* mutant (lack miR-430 activity) embryos at 6 hpf (Fig. 5a) [18, 39]. Our results show that maternal mRNAs containing a miR-430 target seed (GCACTT) that are also highly enriched in either optimal or non-optimal codons are less responsive to miR-430 repression than miR-430 target transcripts possessing average, or neutral, codon optimality (Fig. 5a).

Second, to explore this observation further, we estimated mRNA clearance (log_2_-fold change during MZT) during the MZT with a generalized additive model [41]. This model estimates the log_2_-fold change during the MZT (6 hpf/2 hpf, Additional file 1: Fig. S1a-c) as a non-linear function of codon optimality (Additional file 1: Fig. S4) and miR-430 presence in the 3′UTR. In agreement with the MZ*dicer* data (Fig. 5a) [39], miR-430 predicted repression (miR-430 component) was lower for mRNAs highly enriched in optimal or non-optimal codons (Fig. 5b, *y*-axis), consistent with the strongest microRNA repression being associated with transcripts possessing average or neutral optimality. Similar results were observed in *Xenopus* data for miR-427 (Additional file 1: Fig. S7a) [33].

Third, in our reporter experiment in human cell lines (Fig. 4e), we observed that miR-17 repression activity was affected in a similar manner by codon composition. Specifically, a slightly optimal reporter (scoring within 64% quantile of endogenous transcriptome optimality) was more strongly repressed by microRNA targeting (without microRNA seed vs with microRNA seed) (fold change = 1.6, *p* = 2.10 × 10^−11^, paired *t* test), when compared to a strongly non-optimal reporter (scoring within 22% quantile of endogenous transcriptome optimality) (fold change = 1.3, *p* = 4.14 × 10^−06^, paired *t* test) (Fig. 4e). Thus, reporters in human cells possessing moderate optimality are more responsive to miR-17 repression that reporters highly enriched in non-optimal codons (Fig. 4e).

Fourth, to exclude the possibility that other regulatory elements or mRNA features might account for the differences observed between reporters, we analyzed the stability of a massive reporter library during the zebrafish and *Xenopus* MZT [22]. This library was made by cloning ~ 300–500 length nucleotide sequences derived from the fragmentation of the zebrafish transcriptome into common 5′ and 3′UTRs containing Illumina sequences (Fig. 5c and Additional file 1: Fig. S7b) [22]. Due to the random nature of the fragments, this library possesses coding sequences that differ in composition while sharing a common 5′ and 3′UTR sequences, thereby enabling an unbiased analysis of codon optimality (Additional file 1: Fig. S7b) [22]. However, the vast majority of library fragments contain “premature-stop” codons (e.g., out-of-frame coding fragments or 3′UTR fragments), and therefore, possess “extended” random 3′UTR sequences (Fig. 5c and Additional file 1: Fig. S7b). Taking advantage of this feature, we analyzed the sequences of the library after 2 and 8 hpi into zebrafish embryos and 1 and 9 hpi into *Xenopus* embryos [22]. From all the sequenced reporters (~ 2.2 million unique sequences) [22], we selected those containing stop codons in the variable coding region beyond 350 nucleotides (> 116 codons) and a variable 3′UTR of at least 75 nucleotides. This provided 61,981 and 61,388 unique sequences at 2 and 8 hpi respectively in zebrafish embryos, and 23,927 and 52,016 unique sequences at 1 and 9 hpi respectively in *Xenopus* embryos. Next, we predicted the stability of these reporters based solely on the coding sequence using our model (Fig. 1a). Compared to endogenous genes, our collection of reporters shared a similar distribution of predicted stability profiles (Additional file 1: Fig. S7c). As expected, the libraries at later time points (8 hpi in zebrafish and 9 hpi in *Xenopus*) were enriched with fragments predicted to be more stable, supporting the codon optimality effect [22] and the robustness of our model (Additional file 1: Fig. S7d). We also observed that reporters possessing the miR-430/-427 seed (GCACTT) within the 3′UTR were depleted in the later time point compared to the early time point (*p* < 2 × 10^−10^, binomial regression) (Additional file 1: Fig. S7e), implying that miR-430/-427 can regulate the injected reporter sequences. Therefore, we compared the depletion of the miR-430/-427 seed (GCACTT) across the range of codon optimality levels in the library using bootstrap replicates [40] (Fig. 5d, e). Interestingly, we observed that the miR-430/-427 seed depletion was always consistently stronger for mRNAs with neutral or average codon optimality (Fig. 5d, e). Consistent with the endogenous genes (Fig. 5a, b and Additional file 1: Fig. S7a) and individual reporters (Fig. 4e), these results, using massive reporter libraries in zebrafish and *Xenopus*, support the hypothesis that microRNA activity is diminished for target transcripts containing coding sequences highly enriched in either optimal or non-optimal codons.

### miR-430 antagonizes codon optimality during the MZT

**Fig. 6:**
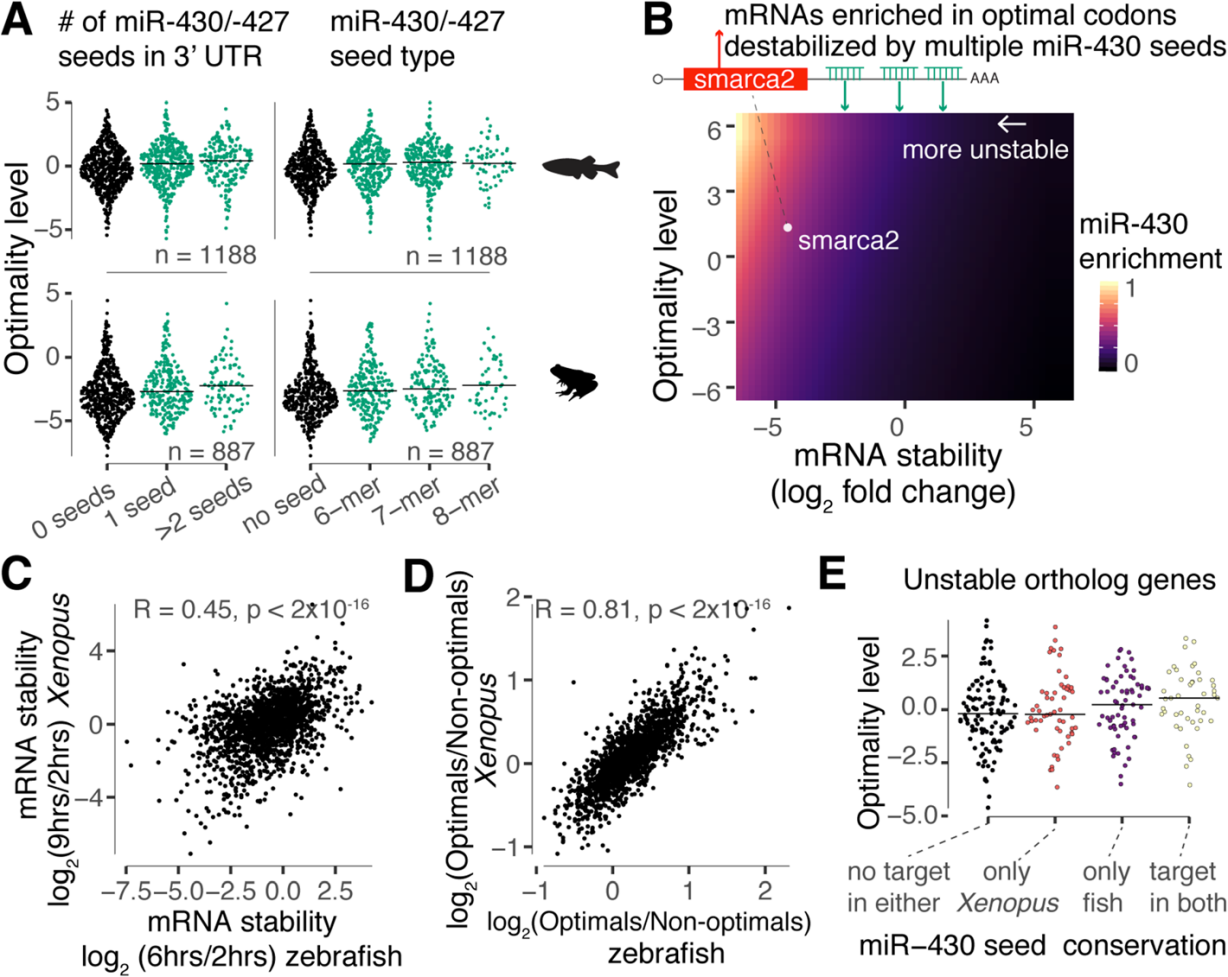
MicroRNAs antagonize codon optimality effect on mRNA stability during the MZT. **a** Sinaplot showing the codon optimality distribution (Table S5) for the top 1000 most unstable maternal genes during zebrafish and *Xenopus* MZT. The stability was defined based on the log_2_-fold change of early (2 h) vs late time points (fish = 6 h, *Xenopus* = 9 h) in fish (Additional file 1: Fig. S1a-b) and *Xenopus* [33]. The genes were divided into groups based on the numbers or seed type of miR-430/427. The content of optimal codons increases with the miR-430 regulation strength (*p* = 2 × 10^−4^ zebrafish, *p* = 2 × 10^−3^ *Xenopus*, linear regression). The *p* value was obtained by comparing the difference in the mean level of optimal codons (PLS1) between genes with and without miR-430/427 sites using a linear model. **b** Heatmap of miR-430 enrichment in the 3′UTR as a function of mRNA stability and codon optimality level. The miR-430 enrichment was estimated with a generalized linear model. Unstable mRNAs enriched in optimal codons temp to contain miR-430 sites (e.g., *smarca2*). **c** Scatter plot comparing the RNA stability, during MZT, of ortholog genes in zebrafish (Additional file 1: Fig. S1a-b) and *Xenopus* [33]. **d** Scatter plot comparing the content of optimal codons in zebrafish and *Xenopus* for ortholog genes [22]. **e** Sinaplot showing the codon optimality distribution for unstable mRNAs in panel **a** that are orthologs (*n* = 280). Messenger RNAs were divided into four categories according to the presence or absence of miR-430/-427 seeds in both species. The orthologous mRNA with miR-430/-427 seeds in both species are the most enriched in optimal codons (*p* = 0.0868, one-way ANOVA test)

Our above results show that repressive *cis*-regulatory elements and codon optimality can operate in conjunction to dictate mRNA stability (Figs. 4 and 5). This combinatorial control may allow transcript stability to be fine-tuned across the transcriptome. Additionally, we posit that microRNAs may also serve as a means to directly antagonize codon-mediated stabilization of the mRNA, likely depending on the number and seed type (8, 7, or 6-mers) as well as in the codon composition (Figs. 4 and 5). To examine this possibility in the context of the MZT, we assessed whether maternal transcripts with inherent stabilizing codon composition (i.e., enriched in optimal codons) are more likely to contain destabilizing 3′UTR regulatory elements (e.g., miR-430 target sites). For both zebrafish and *Xenopus*, we first divided the most unstable maternal mRNAs (top quartile, log_2_-fold change 6 hpf—zebrafish, 9 hpf—Xenopus vs 2 hpf) into three groups based on the number of 6-mer (GCACUU) miR-430/-427 seeds in the 3′UTR (no seed, 1 seed, or > 1 seed) (Additional file 2: Table S1 and Additional file 1: Fig. S1a-b) [33]. We observed that as the number of miR-430/-427 seeds increases, the content of optimal codons (Table S5) also increases (*p* < 0.001, regression analysis) for both species (Fig. 6a). Similar results were obtained when the most unstable mRNAs were divided based on the miR-430/-427 seed strength (8, 7, or 6-mers) (Fig. 6a). We then used a binomial regression model to estimate miR-430 target site enrichment within 3′UTRs across the entire transcriptome. In this model, the enrichment of miR-430 is estimated based on the number of 6-mer seeds in the 3′UTR as a function of the mRNA stability (log_2_-fold change 6 hpf/2 hpf, Table S1) and the content of optimal codons (see the “Methods” section). We also controlled for the 3′UTR length because genes with longer 3′UTRs tend to have more miR-430 seeds and tend to be more unstable [18, 23]. As expected, miR-430 enrichment increases as mRNA stability decreases (Fig. 6b). However, there is a higher miR-430 enrichment for unstable genes enriched in optimal codons (Fig. 6b), supporting the idea that miR-430 antagonizes the codon-mediated stability during MZT in a genome-wide manner. One interesting example is the chromatin-remodeling protein, Smarca2. The *smarca2* transcript plays host to an inherently stabilizing coding sequence yet is effectively degraded due to the presence of multiple 3′UTR miR-430 target sites (Fig. 6b). Interestingly, the expression of *smarca2* without the endogenous 3′UTR (i.e., decoupling codon optimality from microRNA regulation) in zebrafish embryos reduces global heterochromatin establishment in the early embryo [42].

We next tested whether the regulatory coupling of codon optimality and microRNA activity during the MZT is conserved between zebrafish and *Xenopus*. The codons defined as optimal or non-optimal in zebrafish embryos tend to share the same optimality in *Xenopus* embryos [22]. Additionally, orthologous genes tend to share similar contents of optimal codons (*R* = 0.81, *p* < 2 × 10^−16^, Pearson correlation test) (Fig. 6c) and, as expected, tend to display similar stability profiles during MZT (*R* = 0.45, *p* < 2 × 10^−16^, Pearson correlation test) (Fig. 6d) (Additional file 1: Fig. S1a-c; Table S1) [33]. Unstable orthologous transcripts that contained miR-430/-427 target sites in both species displayed a higher ratio of optimal to non-optimal codons compared to those that do not share miR-430/-427 seeds in both or either species (Fig. 6e). Interestingly, this result suggests that transcripts hosting inherently stabilizing coding sequences are under evolutionary pressure to retain destabilizing elements such as miR-430/-427 to ensure robust mRNA clearance during the MZT.

## Discussion

**Fig. 7:**
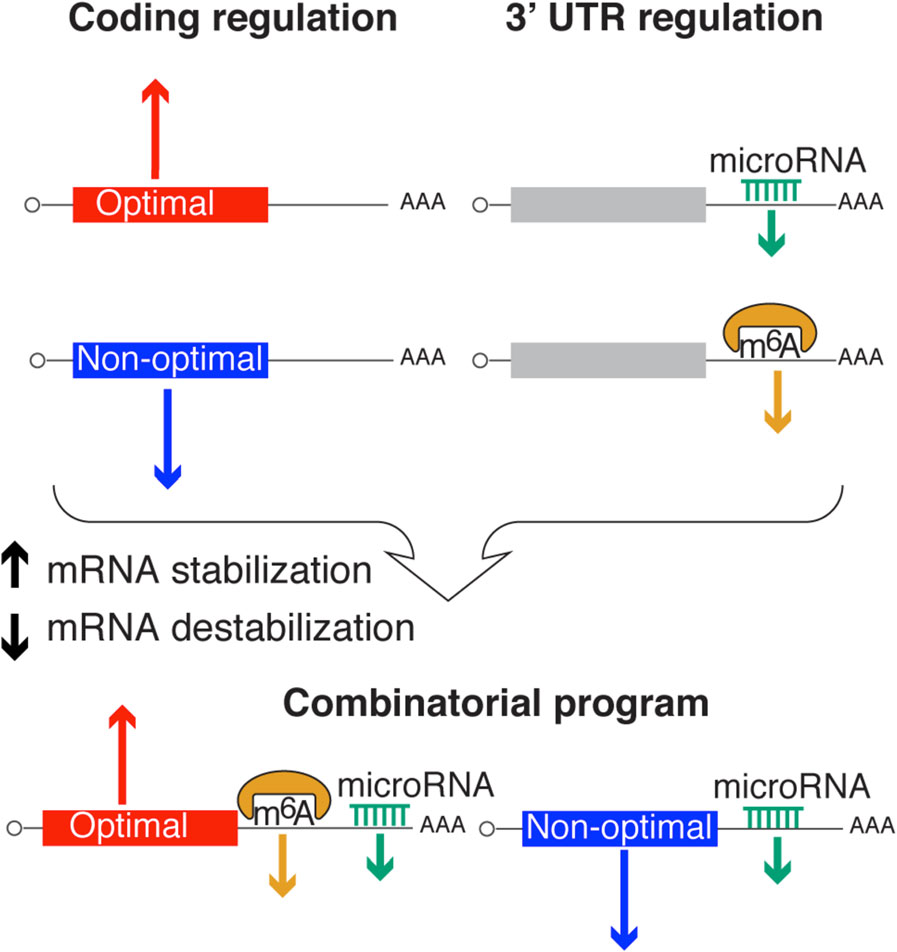
Model showing that the mRNA stability depends on the regulatory elements of the coding and the 3′UTR, suggesting that to fully understand mRNA stability, the regulatory information across the entire mRNA sequence needs to be integrated, rather than focusing solely on the 3′UTR or in the coding sequence

While gene expression is usually attributed to transcription rate, the half-lives of mRNAs strongly affect overall mRNA abundances during homeostasis. Attempts at understanding mRNA stability have primarily focused on *cis*-regulatory elements mainly within the 3′UTR, where a large number of stability determinants (e.g., microRNAs) are known to bind. However, more recent work demonstrates that translation also strongly affects mRNA stability in a codon-dependent manner, indicating that the coding region also contains strong regulatory information [22–25]. Here, we present a computational model to predict vertebrate mRNA stability based on codon composition (Fig. 1). Our model supports the premise that codon composition is the major determinant of mRNA stability in zebrafish and *Xenopus* during early embryogenesis (Fig. 2). The degree to which mRNAs are impacted by microRNAs and RNA methylation (m6A) is also dependent on their respective coding sequences in zebrafish and *Xenopus* embryos, as well as in mouse and human cells (Fig. 4). As such, codon composition can obscure the repressive effects of other regulatory pathways (Figs. 3 and 5). Recently, several studies have aimed to identify novel *cis*-regulatory elements in 3′UTR regions that are active during zebrafish embryogenesis using reporter mRNAs containing a GFP coding sequence [43–45]. Although these methods have been successful in identifying both stabilizing and destabilizing elements in the context of a uniform coding sequence, an accounting of these elements in endogenous transcripts has been largely insufficient to explain stability profiles [43, 44]. We believe that this lack of coherence might be due to the failure to account for codon optimality effects, which impact transcript stability globally. Moreover, we hypothesize that such an accounting will enable the identification of novel regulatory programs resident in the 3′UTR or across the entire mRNA, which may otherwise be obscured by codon composition variability (Figs. 3, 4, 5, and 7).

Our results suggest that miR-430/-427 destabilizing activity can be affected by the coding sequence. The targeting efficacy of miR-430/-427 (zebrafish and *Xenopus*) is stronger in genes with average codon optimality as opposed to genes either highly enriched in optimal or non-optimal codons (Fig. 5). It can be proposed that different regulatory pathways may recruit common mRNA degradation machinery. For example, mRNAs with a very high content of non-optimal codons may already be targeted for degradation at the maximum rate. Accordingly, other pathways, such as microRNAs, cannot increase the mRNA destabilization. Therefore, we posit that it is imperative to consider the coding sequence when attempting to understand the regulatory power of both canonical and non-canonical miRNA sites [20, 46–48].

Our data also indicate that miR-430/-427 can antagonize codon-mediated stabilization during early embryogenesis. Specifically, we find that unstable transcripts enriched in optimal (i.e., stabilizing) codons tend to host miR-430/-427 target sites, suggesting that miR-430/-427 may have been recruited to these mRNAs to counteract the intrinsic “stability” conferred in *cis* by the coding sequence, perhaps to ensure robust degradation of maternal mRNAs during the MZT (Fig. 6). Of course, from an evolutionary point of view, we cannot rule out the possibility that an enrichment in optimal codons is, in fact, an evolved countermeasure in response to the repressive effects of miR-430 and/or m6A (Fig. 6). *Smarca2* is a clear example of a maternal mRNA enriched in optimal codons and containing three miR-430 target sites that serve to destabilize during the MZT [42]. Following from the developmental role and critical clearance of *smarca2* during embryogenesis [42], it will be interesting to dissect the function of maternal mRNAs enriched in optimal codons containing miR-430 target sites. Moreover, it might also be interesting to knock-down maternal mRNAs enriched in non-optimal codons that are actually stable during the MZT [49]. Nonetheless, these results paint a complex picture of how different regulatory mechanisms interact and co-evolve to precisely and temporally modulate mRNA stability.

Future work will aim to understand how the entire mRNA sequence affects stability and how the interplay between codon and *cis*-regulatory mechanisms impacts embryonic development. Studies employing mRNA reporters aimed at further characterizing *cis*-regulatory mechanisms (e.g., microRNAs, RNA modifications, RNA binding proteins) should take into account the degree of codon optimization resident within reporter coding sequences (e.g., “optimized” vs “deoptimized” GFP). Pertinent to this design, our lab has developed a web-interphase, iCodon (ideal codon) [50, 51], to optimize or de-optimize coding sequences based on synonymous mutations. Therefore, iCodon could be used to optimize coding sequences (e.g., Covid-19 vaccines), or to de-optimize reporter sequences such as GFP to study *cis*-regulatory elements in the 3′UTR (e.g., microRNA) using a coding sequence with an average optimality.

Our model predicting gene expression simply takes the codon composition (Fig. 1) and ignores the intrinsic properties of the distribution of the codons across the coding region. For example, the position of the codon can affect codon-mediated mRNA stability in both yeast and zebrafish embryos (Additional file 1: Fig. S2a) [23, 30]. Therefore, in the future, it will be interesting to uncover the grammar/rules of codon-mediated regulation, including the relative positioning (5′ vs 3′) (Additional file 1: Fig. S2c) and ordering of codons. We have recently shown that codon-mediated regulation is dependent on translation initiation rate [24], which in turn is influenced by sequences residing in the 5′ and 3′UTRs as well as cell conditions (e.g., viral infection) [52]. Moreover, translation of small ORFs in the 5′UTR (uORF) as well as in the 3′UTR (dORF) can also affect translation of the main ORF [53–55]. Therefore, in the future, it will be interesting to further characterize the regulatory roles of 5′ and 3′UTRs in shaping translation efficiency as it relates to the codon optimality mechanism. In addition, the availability of tRNAs and charged amino acids has been associated with translation efficiency and mRNA stability in vertebrates [12, 22, 24, 31, 56]. As such, it will be important to integrate tRNA profiles into our model predicting mRNA stability. In sum, our work illuminates the complex crosstalk between *cis*-regulatory pathways and coding sequences in shaping mRNA stability, as well as highlights the need to develop models that integrate both components. Such models will provide valuable insights into early embryonic development, as well as identify underlying causes for gene misregulation in human disease.

## Conclusions

For decades, research on mRNA stability and specifically, on mRNA clearance during developmental transitions, has been focused predominantly on *cis*-regulatory elements that reside outside the coding sequence, mostly within the 3′UTR. This work lays the foundation for exploring mRNA stability regulation from a conceptually different angle: as residing within both coding sequences and 3′UTRs. Therefore, to gain a comprehensive understanding of mRNA stability, we must integrate the regulatory information that exists across the entire mRNA sequence, not just within the 3′UTR or coding sequence. This work highlights the need for researchers studying microRNAs, RNA methylation, RNA binding proteins, and other forms of post-transcriptional regulation to consider how codon composition interacts with these other mechanisms, both within the embryo as well as within other cellular contexts.

## Methods

### Zebrafish early development transcriptome

Three RNA-seq profiles were generated: two (poly(A)-selected and ribosomal RNA-depleted) under normal conditions and one (poly(A)-selected) and treated with alpha-amanitin (Additional file 1: Fig. S1a). For the normal condition profiles, developmentally staged embryos were collected every hour (from 0 to 8 hpf) under normal developmental conditions. After extraction, total RNA was poly(A)-selected by reverse transcription using oligo dT or ribosomal RNA-depleted using Ribo-Zero (Illumina) for RNA-seq library preparation (NextSeq Total RNA, Illumina, USA) according to the manufacturer’s instructions. The injected set of staged embryos were collected every 30 min (from 2 to 7.5 hpf). In this case, 1 nl of alpha-amanitin (200 ng/μl) was injected into single-cell stage embryos to inhibit zygotic transcription via inhibition of polymerases II and III. After extraction, total RNA was poly(A)-selected by reverse transcription using oligo dT for RNA-seq library preparation (NextSeq Total RNA, Illumina, USA), according to the manufacturer’s instructions. Raw reads were demultiplexed into Fastq format, allowing up to one mismatch using Illumina bcl2fastq2 v2.18. Reads were aligned to the UCSC genome danRer11 with STAR aligner (version 2.7.3a), using Ensembl 98 gene models. Gene quantifications were generated using RSEM (version v1.3.0).

In the alpha-amanitin-treated embryos, RNA-seq showed successful inhibition of zygotic gene expression (Additional file 1: Fig. S1b), and a principal component analysis confirmed that time post-fertilization was the main factor of variability between samples (Additional file 1: Fig. S1c). Additionally, early time points recapitulated the known characteristics of mRNA dynamics during zebrafish embryogenesis [22, 44, 57, 58], as was shown by the difference in mRNA levels between poly(A)-enriched RNA and total RNA (Ribo-Zero). The increase in observed poly(A)-tailed transcripts over time reflect expected post-fertilization polyadenylation activity (Additional file 1: Fig. S1d).

### Estimation of mRNA stability

To estimate the stability of the mRNA for each gene, we used a first-order reaction model. mRNA decay rates were determined with the following linear model: *log RNA* = *α* + *β* ∗ *time*. “*Log RNA* ” (logarithm of transcripts per million) measures gene expression after transcriptional blocking by alpha-amanitin treatment. The “*α*” parameter is the model intercept. The parameter “*β*” represents the mRNA decay rate, with negative values corresponding to unstable genes and positive values to stable genes (Additional file 1: Fig. S1e and S1f).

### Prediction of mRNA stability with codon content

#### Data allocation

Besides our mRNA stability profile during early embryogenesis (Figure S1), additional mRNA stability profiles for *Xenopus* [22], zebrafish [22], mouse [29], and human [24] were used. We modeled the mRNA decay rates as a function of codon frequencies and 3′UTR length (Fig. 1a). In total, 68 features were used as predictors: 3 categorical features (species, cell type, and experimental technique) and 65 numerical features (64 codon frequencies and 3′UTR length). The data set was split into training (*n* = 67,817) and testing (*n* = 7536) (Tables S3 and S5). In some cases, the same genes have multiple mRNA stability measurements from different cell types (e.g., human); consequently, the genes selected for the training set were not included in the test data.

#### Data pre-processing

The mRNA decay rates for each profile were standardized (*z*-score normalization) to remove differences in units across experiments (Additional file 4: Table S3). For the predictive features, we employed the following pre-processing pipeline: categorical variables (species, cell type, and experimental technique) were one-hot encoded.

The codon frequencies were calculated from the transcriptome; for each gene, the longest coding isoform was used (Additional file 8: Table S7). The coding sequences and 3′UTR sequences were downloaded from Ensembl. Codon frequencies for each gene were individually normalized to unit norm and gene lengths were log-transformed (3′UTR and coding length). Some genes (5.4%) were missing the 3′UTR sequence, so missing 3′UTR lengths were imputed with the mean across all the genes. For some models (linear, lasso, elastic net, and partial least squares), we generated new predictors consisting of all polynomial (degree 2) combinations of the features to capture possible non-linear effects. Finally, all predictors were standardized (*z*-score normalization).

#### Model evaluation

We evaluated the following machine learning models: ordinary least squares linear regression, elastic net, lasso, PLS regression, AdaBoost, decision tree regressor, k-Nearest Neighbors, gradient boosted decision trees, and random forest (Additional file 1: Fig. S3a). To evaluate each model, we used the *R*^2^ score as the performance metric. We employed grouped-5-fold cross-validation to tune and evaluate the models. Grouped-5-fold cross-validation ensures that the same gene is never in the training and testing data simultaneously due to multiple measurements for the same gene. In the preliminary analysis, we observed that other cross-validation methods produce overfitting models. In addition, we performed a learning curve analysis that showed that the performance increases with the size of the training data (Additional file 1: Fig. S3b). Hence, combining the species data helps to capture the signal from the noise. Finally, we selected the lasso model [28] as the final model to predict mRNA stability. All steps of the modeling were performed in Python (v = 3.6.8) using the scikit-learn (v = 0.20.3) machine learning library [59].

#### Evaluation of codon position effect in mRNA stability prediction

To interrogate whether the position of the codons across the ORF has different codon-mediated effects on mRNA stability, we generated sliding windows for each endogenous mRNA. Each sliding window represents a proportion (15%) of the total mRNA sequence. In total, 50 windows were used covering the complete ORF sequence. Each of these sliding windows represents a unique position for each mRNA. For each of these sliding windows, the frequency of the codons in the window was computed. Using the codon frequencies and the mRNA decay rates, for zebrafish, we computed the codon stabilization coefficient (CSC) relative to the position of each window (Additional file 1: Fig. S2a). To evaluate the effect of the codon position in predicting mRNA stability in zebrafish (Additional file 3: Table S2), five linear models were trained using the codon frequencies at different positions along each transcript sequence (Additional file 1: Fig. S2b). Three of these models used only one-third of the transcript region (5′ end, middle, and 3′ end), a model used all the coding region, and a final model included the three regions (5′ end, middle, and 3′ end). For instance, for the codon CAC, the model contained three variables for CAC indicating the frequencies of the CAC codon in each of the three regions. The predictive performance for each of these models was estimated using the *R*^2^ score.

### Optimal/non-optimal reporters in zebrafish embryos

To assess the effect of optimality in zebrafish embryos, in vitro transcribed mRNA encoding for mCherry (40 ng) coupled to either an optimal or a non-optimal sequence by P2A was co-injected into one-cell stage zebrafish embryos with and GFP mRNA (50 ng) as a control [24]. Injected embryos were incubated in the dark at 28 °C. At 8 hpi, embryos were imaged first for red and then green fluorescence using a stereo fluorescent microscope. Fluorescence intensity was measured using ImageJ.

#### Measuring codon optimality at the gene level

Although the codon stabilization coefficient has been defined as a measure of codon optimality codon-wise [22, 24, 25, 60], we lack a reliable measure for codon optimality at the gene level. Previous studies have used the proportion of optimal codons as a gene-wise measure of optimality [22, 24, 25]. We took advantage of the growing number of mRNA stability datasets that have been published [22, 24, 29].

Our codon optimality measure (Additional file 1: Fig. S4a) was derived using a supervised dimensional reduction technique, partial least squares (PLS) [61], that finds components that maximally summarize the variation of the codon content while simultaneously requiring these components to have a maximum correlation with the mRNA stability profiles [62]. The codon frequencies (64 dimensions) are projected in a 2-dimensional space (Additional file 1: Fig. S4b); hence, each gene is represented as two components (PLS). These components correlated with the proportion of optimal codons (Additional file 1: Fig. S4c) and the mRNA decay rates (Additional file 1: Fig. S4d). This correlation suggests that our codon optimality assessment represents a good alternative to measuring codon optimality gene-wise.

#### Model comparison during the MZT

To compare the regulatory effect of codon optimality with other known regulatory modes (i.e., microRNAs), we used a Bayesian model comparison (Fig. 2c). We fit the following linear models using the brms package [63]:

$$ (1)\ {\log}_2 fold\ change\sim Normal\left(\alpha +{\beta}_1 PL{S}_1+{\beta}_2 PL{S}_2,\sigma \right) $$

$$ (2)\ {\log}_2 fold\ change\sim Normal\left(\alpha +{\beta}_1 Seeds,\sigma \right) $$

$$ (3)\kern0.5em {\log}_2 fold\ change\sim Normal\left(\alpha +{\beta}_1m6A,\sigma \right) $$

The “log_2_*fold change*” variable is the log_2_ fold change of mRNA levels between early stage (zebrafish 2 hpf and *Xenopus* 1 hpf) and shield stage (zebrafish 6 hpf and *Xenopus* 9 hpf). For zebrafish, we used our RNA-seq profile during MZT, and for *Xenopus*, we used published RNA-seq [33]. For the codon optimality model [1], *PLS*_1_ and *PLS*_2_ represent the content of optimal codons in endogenous genes (Figure S4). In the microRNA model [2], *Seeds* is the number of miR-430/-437 seeds (6-mer GCACUU) in the 3′UTR. In the m6A model [3], *m*6*A* is an indicator of whether or not the gene is an m6A target [10]. We computed the approximate leave-one-out cross-validation using Pareto smoothed importance sampling [32] for each model. Model weights were calculated via the stacking of predictive distributions [64].

#### MiR-430 enrichment model

To estimate the enrichment of miR-430 seeds (6-mer GCACUU) in the 3′UTR (Fig. 6b), we used the following Bayesian binomial regression model:

$$ seeds\sim Binomial\left(n= length\ {3}^{\prime } UTR,\kern0.5em p={p}_i\right) $$

$$ logit\left({p}_i\right)=\alpha +{\beta}_1 foldchange+{\beta}_2 PL{S}_1+{\beta}_2 PL{S}_2 $$

The *foldchange* variable corresponds to the log_2_-fold change in mRNA levels between 6 and 2 hpf (Additional file 1: Fig. S1a-c). *PLS*_1_ and *PLS*_2_ correspond to the content of optimal codons (Additional file 1: Fig. S4). We used the weakly or non-informative default priors set in the package brms [63].

For all of the Bayesian models used (Fig. 2c and 6b), we ran four MCMC chains simultaneously, each with 2000 iterations and a warmup of 1000 iterations. We validated all chain convergence visually.

#### MicroRNA experiments with optimal and non-optimal reporters

To clone the reporters used for mammalian transfection, PCR fragments were amplified with Q5 polymerase (NEB), followed by restriction cloning using reagents from NEB according to the provided instructions. For transfection, 293T cells (passage < 20) obtained from the Tissue Culture core facility from the Stowers Institute for Medical Research were cultured with DMEM media supplemented with 10% FBS. Cells were transfected in a 96-well plate at 70–80% confluency with Lipofectamine 3000 (Thermo Fisher Scientific), following the manufacturer’s instructions. Twenty-four hours after transfection, cells were detached with trypsin and suspended in DMEM media. The median of the fluorescent reporter intensity of the cells was quantified in a ZE5 flow cytometer (Bio-Rad) using the GFP (488/510) and mCherry (587/610) detector and analyzed with FCS Express 7.

#### Analysis of miR-430/-427 depletion and codon optimality in massive reporter libraries

The reported library transcript sequences, both zebrafish and *Xenopus* embryos, were downloaded from a previous publication [22]. The sequences were mapped with Bowtie2 to zebrafish Ensembl release 80 cDNA (longest transcript per gene) using the following parameters: –local, –no-mixed, –no-discordant, –no-overlap, –norc, –no-unal, -I 200, -X 600 [65, 66]. The fragment sequences spanning the full locus delineated by each pair of reads were extracted using SAMtools and BEDTools [67, 68]. In order to get sequences with variable coding and random 3′UTR sequences, we selected sequences containing a stop codon in the variable coding region. And from those sequences, the ones with a variable coding region of at least 117 codons and a variable 3′UTR region of at least 75 nucleotides were selected. Next, sequences that were represented more than 5 times were discarded. Using the variable coding region, we estimated the codon optimality level for each sequence as the predicted mRNA stability with our model (Fig. 1a). The presence of the miR-430/-427 seed (GCACTT) was computed from the variable 3′UTR region. The replicates for each species and time point were merged. For the analysis, the reporter library was divided into 7-tiles of equal size and increasing codon optimality level; for each of these tiles, the log_2_-fold change of the miR-430/-427 seed depletion was estimated by comparing the occurrence of seed between the late vs. early time points. The statistical significance of the miR-430 depletion pattern was estimated with bootstraps replicates [40].

All statistical analyses were performed in *R* (version = 3.6.2). The source code for the analysis presented in this study is available from GitHub [69].

## Supplementary Information

**Additional file 1: Supplementary figures**.

**Additional file 2: Table S1**.

**Additional file 3: Table S2**.

**Additional file 4: Table S3**.

**Additional file 5: Table S4**.

**Additional file 6: Table S5**.

**Additional file 7: Table S6**.

**Additional file 8: Table S7**.

**Additional file 9:.** Review history.

## Data Availability

Sequencing data have been deposited in the NCBI Gene Expression Omnibus, GSE148391 [69]. Data were collected, stored, and preserved including analysis code using the Git version control software in combination with off-site storage and hosting website GitHub. The code used to generate figures and analyses are available on the GitHub repository [70] and Zenodo [71], where the scientific community is invited to visit and open constructive issues. The code is distributed under the MIT License. The following previously published data sets were used: NCBI Gene Expression Omnibus ID GSE126523, mRNA decay profiles for human cells [24] NCBI Gene Expression Omnibus ID GSE99978, mRNA decay profile in wildtype mES cells [29] NCBI, Sequence Read Archive (SRA) SRP072296, mRNA decay profile during MZT for zebrafish and *Xenopus* [22] NCBI Gene Expression Omnibus ID GSE65785, gene expression dynamics in *Xenopus* [33]
