# Supplementary material for: Crosstalk between codon optimality and cis-regulatory elements dictates mRNA stability
Source: Genome Biol. 2021 Jan 5;22:14. doi: 10.1186/s13059-020-02251-5 (PMC7783504; doi:10.1186/s13059-020-02251-5)
Supplement: Supplementary file 1 — Additional file 1: Supplementary figures. [file 13059_2020_2251_MOESM1_ESM.pdf]

Fig S1, Medina et al

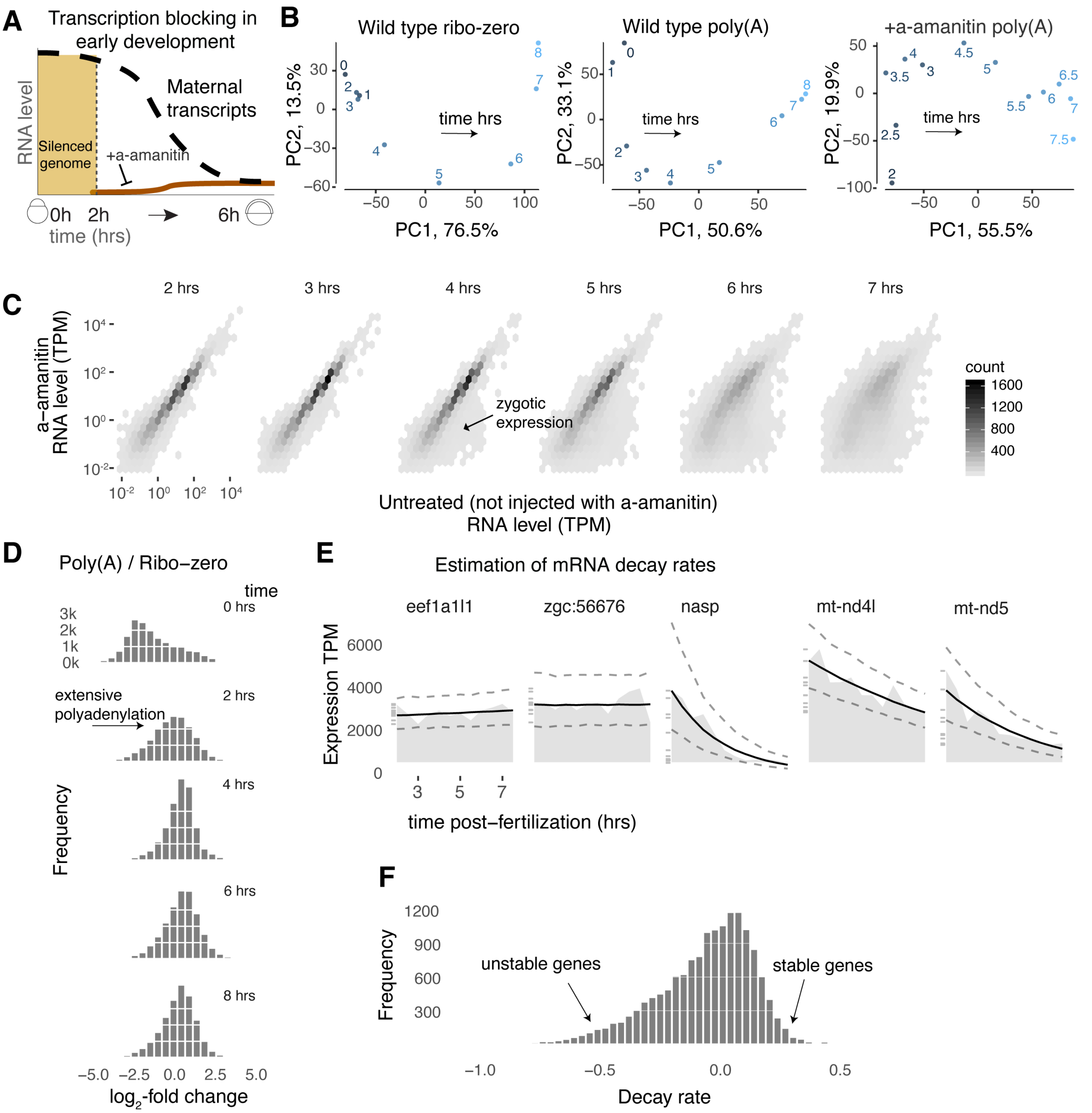

Supplemental figure 1. Accessing mRNA dynamics during MZT in zebrafish. (**A**) Diagram of mRNA level during early embryogenesis in zebrafish embryos injected with α-amanitin. Embryos were treated with α-amanitin to inhibit zygotic gene expression. Samples were collected every 30 minutes for mRNA sequencing (Table S1). (**B**) Principal Component Analysis of mRNA-seq profiles. Each dot is an mRNA-seq experiment, and the number shown is the time after fertilization. The major component of variation correlates with the time post-fertilization. (**C**) Bivariate density comparing the mRNA levels of the α-amanitin treated embryos and untreated embryos. Zygotic gene expression is observed at approximately 4 hours post fertilization (hpf) (Table S1). (**D**) Distribution of foldchange ($\log_2$ ratio) of mRNA levels between poly-(A) enriched RNA and total RNA (Ribo-Zero). (**E**) Scatter plot showing the mRNA level and time post fertilization for some genes after α-amanitin treatment. The slope of the black line, in the log scale, corresponds to the mRNA decay rate. (**F**) Distribution of mRNA stability decay rates estimated using a first-order reaction model on the α-amanitin RNA-seq profile (Table S2).

Fig S2, Medina et al

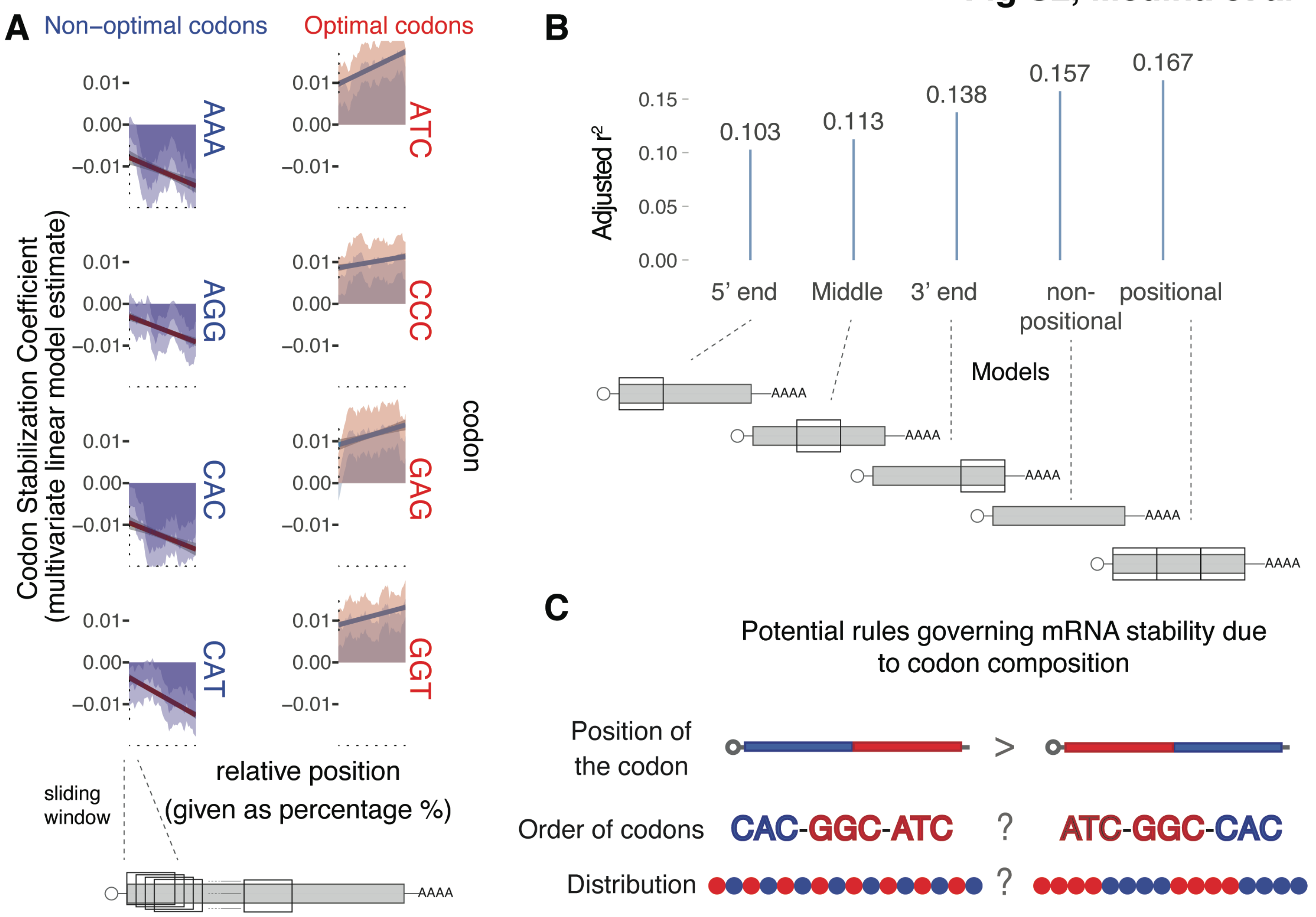

Supplemental figure 2. The codon effect on mRNA stability is more influential in the 3' than the 5' region of the ORF.

(A) Scatter plot comparing the Codon Stabilization Coefficient (CSC) and gene position for few codons in zebrafish embryos. Codons on the left grid are non-optimal, and codons on the right are optimal (Bazzini et al., 2016). The CSC was estimated in different positions of the transcript with a sliding window. The CSC increases (absolute value) as the sliding window approaches the 3' end. This result indicates that codon optimality regulation is more robust in the 3' end. **(B)** We trained five linear models to predict mRNA stability in zebrafish. The bar plot compares the adjusted r-squared values: the "5' end" model contains the first third of the codons in the coding sequence, the "middle" model includes the second third, the "3' end" model covers the last third, the "non-positional" model contains all the codons without indicating the position, and the "positional" model includes the codons and also indicator variables for the position (5' end, middle, and 3' end). The diagram depicts the codon regions of a gene that were used as predictors to train the models. **(C)** Potential grammar/rules of codon optimality regulation.

Fig S3, Medina et al

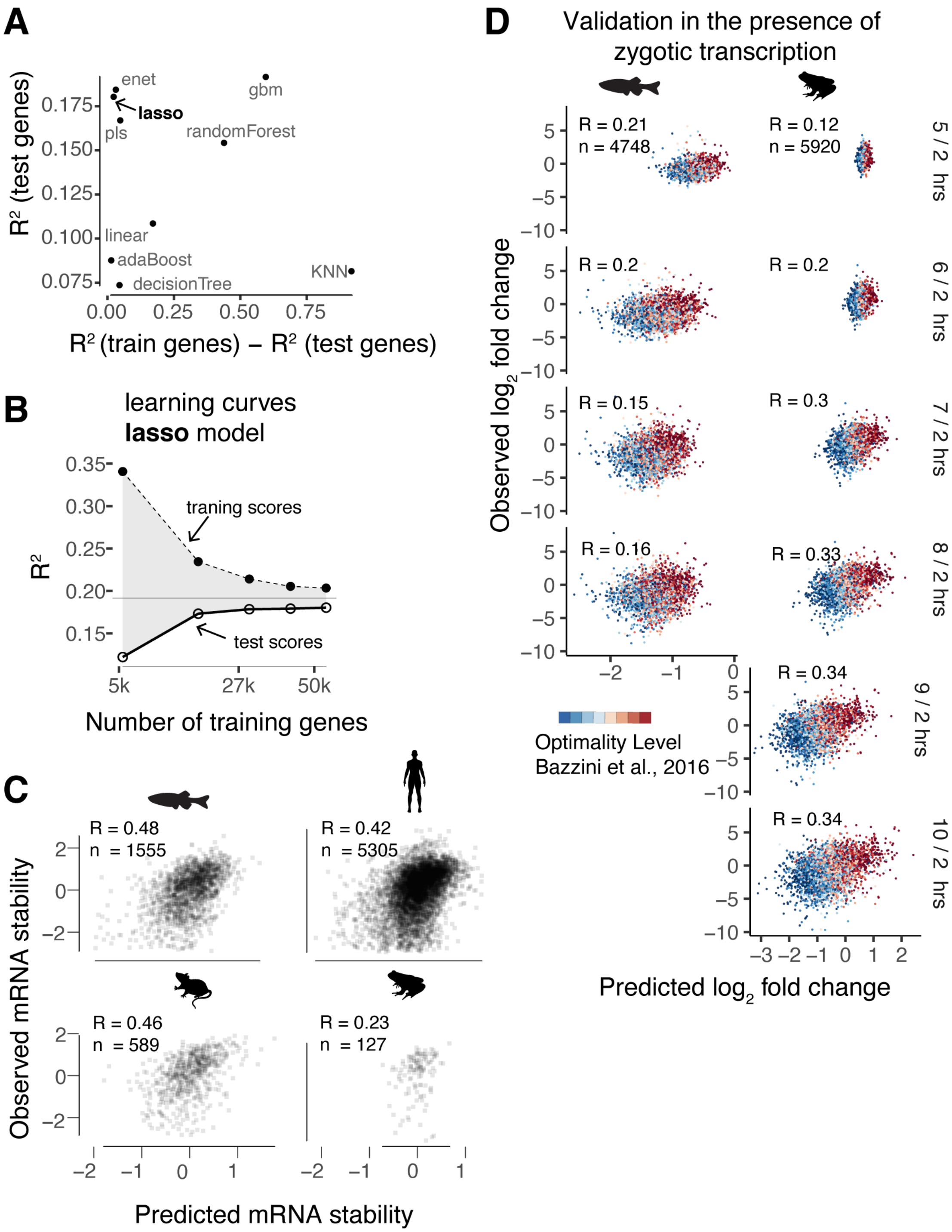

Supplemental figure 3. Predictive model of mRNA stability based on codon content. (**A**) Different types of machine learning models were tested. The y-axis shows the explained test data variation (R^2 score) and the x-axis shows the overfitting amount as measured by the difference between training and testing R^2 scores. The lasso model was selected as the final model for predicting mRNA stability. (**B**) Learning curve for lasso model. The gray area shows the overfitting amount. Overfitting decreases with the addition of training genes. (**C**) Predicted vs. observed mRNA stability for genes in the test data set across species ($p < 0.0084$, Pearson correlation test)**.** (**D**) Scatter plots of observed vs. predicted mRNA stability dynamics, during MZT, for maternal mRNAs in zebrafish (Additional file 1: Fig. S1a-c) and *Xenopus* (Owens et al., 2016). Each panel represents a different time point, as indicated on the right side. The observed mRNA stability is the expression level at each timepoint normalized to 2 hpf ($\log_2$-fold change X/2 hpf). Using the predicted decay rate, we fit a linear model to estimate the $\log_2$-fold change in the presence of zygotic transcription. The R value shown corresponds to the correlation between the prediction and the observed $\log_2$-fold change and it was computed with a Pearson correlation test (all $p < 2x10^{-16}$). The gradient of color represents the content of optimal codons (Bazzini et al., 2016).

Fig S4, Medina et al

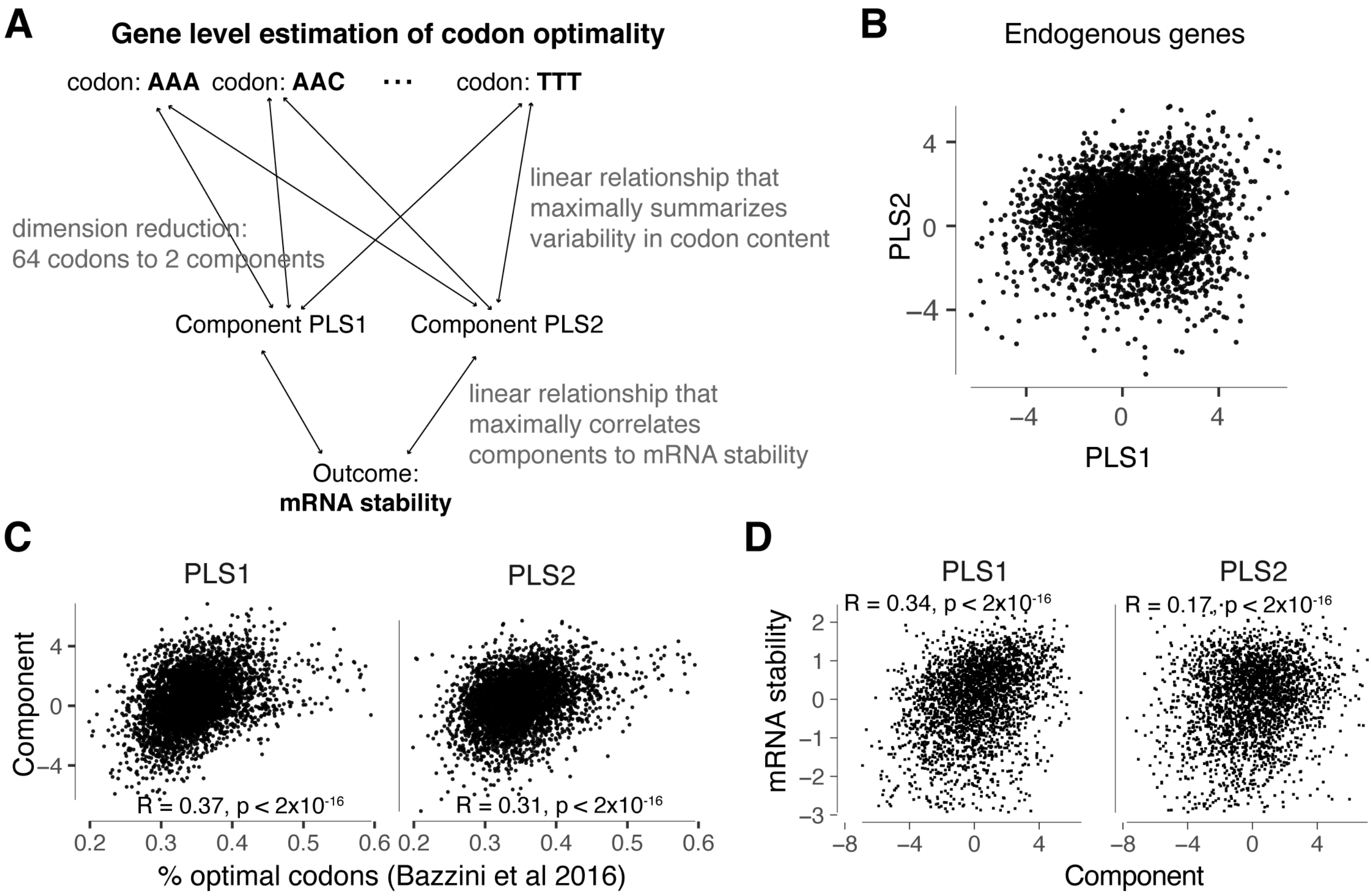

Supplemental figure 4. Gene level measurements of codon optimality in endogenous genes. (**A**) A diagram depicting the structure of the Partial Least Squares (PLS) model used to measure codon optimality in endogenous genes (Table S5). The PLS model projects the 64-dimensional codon space into 2 dimensions (PLS1 and PLS2), figure adapted from Fig 6.9 in (Kuhn & Johnson, 2013). (**B**) Scatter plot visualizing the endogenous genes, in zebrafish (n = 4737), as the projection of the two principal components. Each gene is represented in a two-dimensional space with two components PLS1 and PLS2. (**C**) PLS components correlate with the proportion of optimal codons in zebrafish. Pearson correlation test $p < 2.2 \times 10^{-16}$. (**D**) Scatter plot matrix comparing mRNA stability (decay rates), and PLS components in zebrafish (n = 4737). Pearson correlation test, $p < 2.2 \times 10^{-16}$.

Fig S5, Medina et al

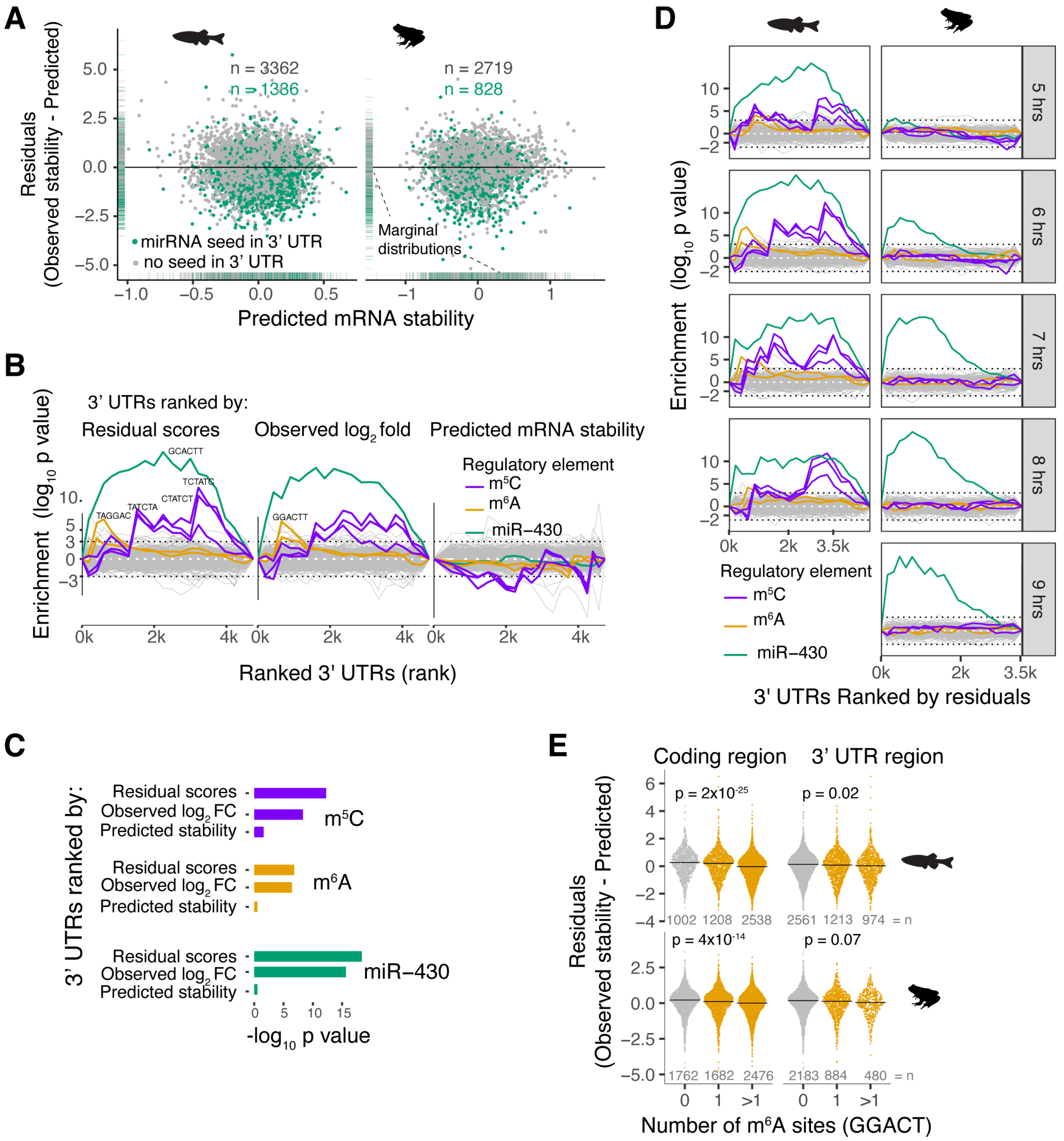

Supplemental figure 5. Residual values uncover microRNA regulation (**A**) Scatter plot of residual scores (Table S4) and predicted mRNA stability during the MZT for zebrafish and *Xenopus*. Messenger RNAs with miR-430/-427 seed (GCACUU) are highlighted in green color. The rugs on the sides represent the marginal distributions. (**B**) Sylamer enrichment landscape plots (Van Dongen, Abreu-Goodger, & Enright, 2008) for 6-mers words in zebrafish genes ranked by 1) residual scores (left) (Table S4), 2) wild-type stability ($\log_2$ fold change 6 hrs / 2 hrs, center) (Additional file 1: Fig. S1a-c), and 3) predicted mRNA stability (right) (Table S4). The x-axes represent the sorted gene list from negative to positive. The y-axes show the hypergeometric significance for each 6-mer at each leading bin. Positive values indicate enrichment ($-\log_{10}$ p value) and negative values, depletion ($\log_{10}$ p value) (Van Dongen et al., 2008). The 6-mers associated with regulatory elements during MZT are color-coded (green miR-430, yellow m6A, and purple m^5C). (**C**) Bar-plot comparing the enrichment of 6-mer signals ($\log_{10}$ p value) associated with mRNA stability regulation during MZT in zebrafish (Additional file 1: Fig. S1a-c). In all the cases the enrichment is higher when 3′UTRs are ranked according to residual scores. This result indicates that the unexplained variation by codon optimality highlights regulatory signals in the 3′UTR or coding regions. There is virtually no enrichment when 3′UTRs are ranked by codon predicted stability, indicating that the model predictions are not derived by 3′UTR sequence but by the codon composition. (**D**) Same as B, but 3′UTRs are always ranked by residual scores at different time points in zebrafish and *Xenopus*. (**E**) Sinaplot showing the distribution of the residual scores for genes containing the putative m6A recognition site GGACT (Zhao et al., 2017). The genes are divided into three groups according to the number of m6A sites, the left grid represents the coding sequence and the right is the 3′UTR sequence. The m6A degradation signal is more influential in the coding sequence than in the

3'UTR. The p values shown were computed with a linear model and correspond to the coefficient that represents the number of m6A sites. The grey numbers on the bottom represent the number of genes in each group.

Fig S6, Medina et al

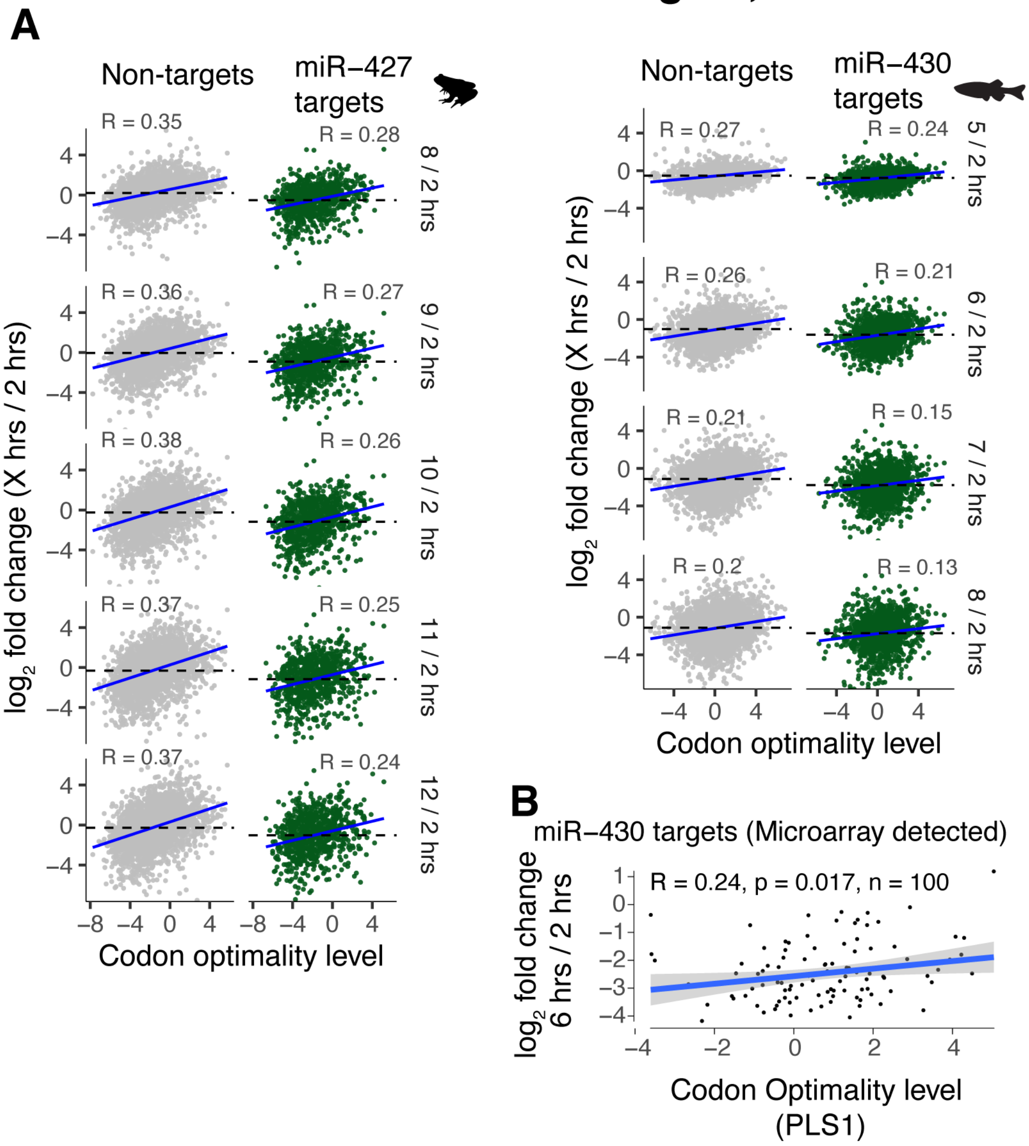

Supplemental figure 6. Codon optimality and microRNAs act additive to regulate gene expression. (**A**) Scatter plot of codon optimality level (x-axis) and expression level at each time point, expression normalized to 2 hpf (log2 fold change X/2 hpf), for maternal genes, in *Xenopus* (Owens et al., 2016) and zebrafish (Additional file 1: Fig. S1a-b). The left grid shows the genes, grey color, that do not contain miR-430/-427 seed site in the 3′UTR, genes on the right, dark green, contain the 6-mer seed (GCACTT) in the 3′UTR. The dashed black line shows the average of each group. The dashed line on the right is lower compared to the left grid, this indicates the destabilization conferred by miR-430/-427. The blue line is the best fit linear regression line ($p < 0.0001$). The R value shown corresponds to a Pearson correlation test, all $p < 1.2x10^{-6}$. **(B**) Scatter plot showing the level of optimal codons (Table S5) vs. the log_2-fold change expression (6 hrs / 2 hrs) for validated miR-430 targets in zebrafish (Giraldez et al., 2006).

Fig S7, Medina et al

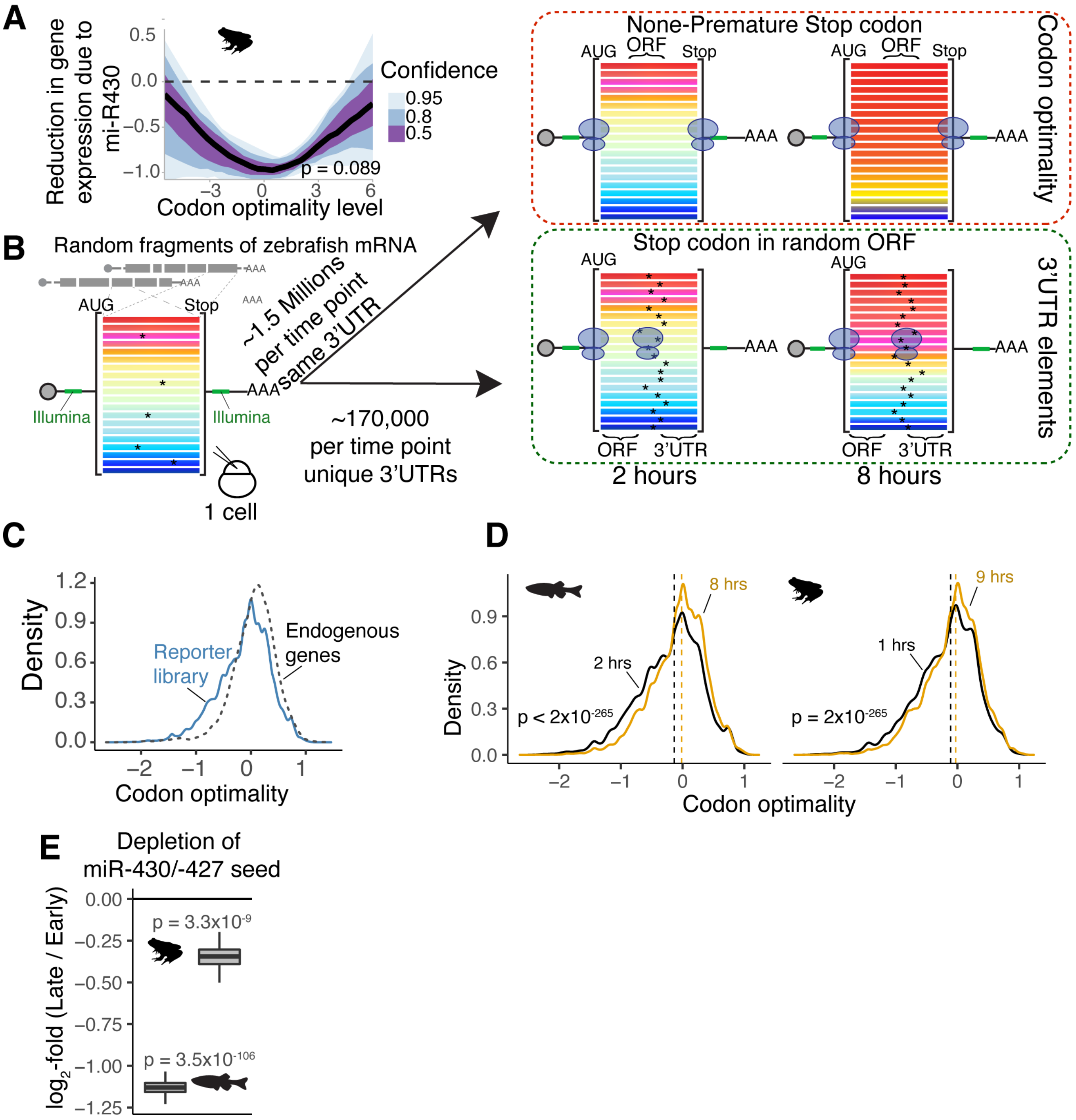

Supplemental figure 7. Codon content affects microRNA targeting efficacy. (**A**) Line plot showing the expected decrease in gene expression due to miR-427 during *Xenopus* MZT ($\log_2$-fold change 9 vs 2 hrs) (Owens et al., 2016). The y-axis represents a measure, estimated from the data, of the miR-427 repressive strength with respect to codon optimality (x-axis). For example, for an mRNA that is very repressed by miR-427, the miR-427 component will be larger (higher negative value in the y-axis). However, for another gene, where the miR-427 repression is weak, the miR-427 component is smaller (closer to 0 in the y-axis). The p value denotes the statistical significance of the non-linear interaction between codon optimality and miR-430 presence (F-test) obtained with a generalized additive model (Hastie & Tibshirani, 1990). The confidence interval was determined with bootstrap replicates (n = 100) (Efron & Tibshirani, 1994). (**B**) Scheme of the reporter library which includes random fragments of the zebrafish transcriptome (Bazzini et al., 2016). Transcripts fragments share the same 5′ and 3′UTR but some fragment sequences contain a stop codon. These stop codons create a random and longer 3′UTR sequence. The fragments with no stop codons were previously used to study codon optimality (Bazzini et al., 2016). Messenger RNAs were previously injected at the one-cell stage in zebrafish and *Xenopus*, and the reporter library is analyzed (2 and 8 hrs post-injection in fish and 1 and 9 hrs post-injection in *Xenopus*) after high-throughput sequencing. (**C**) Predicted mRNA stability distribution of reporter sequences. The dotted line is the predicted stability of the endogenous genes. This predicted stability was used as a measure of codon optimality for the massive reporter library. (**D**) Codon optimality distribution of the reporter library during early and late time points. In the late time point, the library is depleted in non-optimal sequences and enriched in optimal ones (p value computed with a linear model). (**E**) Boxplot showing the depletion of

miR-430/-427 seed in the massive library between late and early time points in zebrafish and *Xenopus*.

Bazzini, A. A., del Viso, F., Moreno-Mateos, M. A., Johnstone, T. G., Vejnar, C. E., Qin, Y., . . . Giraldez, A. J. (2016). Codon identity regulates mRNA stability and translation efficiency during the maternal-to-zygotic transition. *The EMBO journal, 35*(19), 2087-2103.

Efron, B., & Tibshirani, R. J. (1994). *An introduction to the bootstrap*: CRC press.

Giraldez, A. J., Mishima, Y., Rihel, J., Grocock, R. J., Van Dongen, S., Inoue, K., . . . Schier, A. F. (2006). Zebrafish MiR-430 promotes deadenylation and clearance of maternal mRNAs. *Science, 312*(5770), 75-79.

Hastie, T. J., & Tibshirani, R. J. (1990). *Generalized additive models* (Vol. 43): CRC press.

Kuhn, M., & Johnson, K. (2013). *Applied predictive modeling* (Vol. 26): Springer.

Owens, N. D., Blitz, I. L., Lane, M. A., Patrushev, I., Overton, J. D., Gilchrist, M. J., . . . Khokha, M. K. (2016). Measuring absolute RNA copy numbers at high temporal resolution reveals transcriptome kinetics in development. *Cell reports, 14*(3), 632-647.

Van Dongen, S., Abreu-Goodger, C., & Enright, A. J. (2008). Detecting microRNA binding and siRNA off-target effects from expression data. *Nature methods, 5*(12), 1023-1025.

Zhao, B. S., Wang, X., Beadell, A. V., Lu, Z., Shi, H., Kuuspalu, A., . . . He, C. (2017). m 6 A-dependent maternal mRNA clearance facilitates zebrafish maternal-to-zygotic transition. *Nature, 542*(7642), 475-478.

Crosstalk between codon optimality and *cis*-regulatory elements dictates mRNA stability

Santiago Gerardo Medina-Muñoz, Gopal Kushawah, Luciana Andrea Castellano, Michay Diez, Michelle Lynn DeVore, María José Blanco Salazar, Ariel Alejandro Bazzini

Published data

A short description of each dataset accompanying this publication.

Table S1: zebrafish RNA-seq time course during MZT

RNA-seq gene level quantifications. Raw sequencing data have been deposited in NCBI Gene Expression Omnibus GSE148391. This table contains 35,117 rows and 31 columns. Each row represents a gene.

The column names contains all the relevant sample description:

- **Gene_ID ->** zebrafish ensembl gene id
- **Treatment_x-y_hrs-RNAseq_z** Here each column represents a single RNA-seq experiment containing the gene expression levels (Transcripts Per Million). The variables x, y, and z are placed holders: `x`: Some embryos were treated with alpha-amanitin to inhibit zygotic transcription. This takes only two values: `aamanitin` which denotes alpha-amanitin treated embryos and `wt` which represents untreated embryos. `y`: time post fertilization (hours). `z`: RNA-seq method `ribo` for ribosomal-RNA depletion and `polya` for poly-A selection.

A few rows and columns of the table are shown below:

Table S1

Gene_ID	Treatment_wt-0_hrs-RNAseq_ribo	Treatment_wt-1_hrs-RNAseq_ribo
ENSDARG00000000001	12.02	12.47
ENSDARG00000000068	52.57	48.97
ENSDARG00000000069	219.59	163.14
ENSDARG00000000019	49.06	39.72
ENSDARG00000000002	2.71	2.20

Table S2: mRNA stability during MZT

Messenger RNA degradation rates estimated from the alpha-amanitin time course. For additional information see the paper's methods section: *"Estimation of mRNA stability"*.

95% confidence interval lower limit Columns description:

- **Gene_ID ->** zebrafish ensembl gene id.
- **decay_rate ->** estimated decay rate, negative values indicate unstable genes and positive values stable genes.
- **std.error ->** standard error of the estimate.
- **conf.low ->** lower limit 95% confidence interval.
- **conf.high ->** upper limit 95% confidence interval.

A few rows of the table are shown below:

Table S2

Gene_ID	decay_rate	std.error	conf.low	conf.high
ENSDARG00000000001	-0.4317706	0.0610166	-0.5677241	-0.2958171
ENSDARG00000000018	0.1329993	0.0487225	0.0244389	0.2415597
ENSDARG00000000019	0.0667906	0.0190031	0.0244490	0.1091321
ENSDARG00000000068	0.0057338	0.0202691	-0.0394285	0.0508961
ENSDARG00000000069	-0.3366622	0.0318811	-0.4076978	-0.2656266
ENSDARG00000000086	0.3106115	0.0428094	0.2152261	0.4059968

Table S3: Training/testing data to train machine learning predictor of mRNA stability

Data that was used to train and evaluate machine learning predictor model. This table contains 75,351 rows and 8 columns.

Columns description:

- **gene_id ->** ensembl gene id.
- **specie ->** specie vertebrate (human = *H. sapiens*, fish = *D. rerio*, mouse = *M. musculus*, and xenopus = *X. tropicalis*).
- **cell_type ->** cell type from where mRNA stability measurements were derived.
- **datatype ->** How where mRNA stability mesurements generated?:
  - `endogenous` Actinomycin D was used to block transcription
  - `aamanitin ribo` embryos treated with alpha-amanitin (RNA-seq ribosomal-RNA depletion)
  - `aamanitin polya` embryos treated with alpha-amanitin (RNA-seq poly-A selection)

    - `slam-seq` SLAM-seq.
- **decay_rate ->** mRNA degradation rates (see the note below).
- **utrlenlog ->** 3' UTR length log-transformed.
- **cdslenlog ->** cds length log-transformed.
- **allocation ->** this variable denotes wether the given observations was used for training or for testing (validation). Note: the same gene-id is never in training or testing simultaneously.

Notes:

- The mRNA degradation rates in this table were standardized (mean = 0 and standard deviation = 1). The next table below shows the mean and standard deviations of the original data. By applying the inverse transform, the values in the original scale can be recovered.
- This table is missing the codon composition (codon frequencies). Codons frequencies were computed, for each gene, from the longest coding isoform sequence.

means and strandar deviations

specie	cell_type	datatype	mean_decayrate	stdeviation_decayrate
fish	embryo mzt	aamanitin polya	-0.0629208	0.1860839
fish	embryo mzt	aamanitin ribo	-0.0072156	0.0032341
human	293t	endogenous	0.0051652	0.0432408
human	hela	endogenous	-0.0009781	0.0542492
human	k562	endogenous	-0.0176985	0.0668504
human	k562	slam-seq	-0.1313344	0.0672748
human	RPE	endogenous	-0.0071829	0.0597563
mouse	mES cells	slam-seq	-0.1961037	0.0853733
xenopus	embryo mzt	aamanitin ribo	-0.0016150	0.0007771

Decay rates, for the given specie, were obtained from the following publications:

- zebrafish: This study; AA Bazzini, F del Viso, MA Moreno-Mateos… - The EMBO journal, 2016.
- human: Q Wu, SG Medina, G Kushawah, ML DeVore… - Elife, 2019.
- xenopus: AA Bazzini, F del Viso, MA Moreno-Mateos… - The EMBO journal, 2016.
- mouse: VA Herzog, B Reichholf, T Neumann, P Rescheneder… - Nature …, 2017.

A few rows of the table are shown below excluding the `coding` column:

Table S3

gene_id	speci e	cell_ty pe	datatype	decay_ra te	utrlenl og	cdslenl og	allocati on
ENSG00000013523	huma n	RPE	endogeno us	0.29783 91	8.0232 25	7.6078 78	training

ENSG00000131943	human	k562	slam-seq	-0.8748520	8.249837	6.131227	training
ENSG00000104325	human	293t	endogenous	-0.1878186	7.459915	6.916715	training
ENSG00000227124	human	k562	endogenous	0.0023865	6.916715	7.917901	training
ENSMUSG00000018865	mouse	mES cells	slam-seq	0.6431109	7.225481	6.752270	training

Table S4: Predictions and residual values during MZT for zebrafish and xenopus.

This data is part of **Fig. 2** (see paper). This table contains the codon predicted stability for zebrafish and xenopus during MZT. This table contains 10,668 rows and 4 columns.

Columns description:

- **gene_id ->**: ensembl gene id, the genes here are maternally deposited.
- **specie ->**: either zebrafish or xenopus.
- **residual ->**: residual values, values close to zero indicate that the model predicts well the stability and large values that the model is far from the observed value. The residual is the difference between observed and predicted mRNA stability.

A few rows of the table are shown below:

Table S4

gene_id	specie	predicted	residual
ENSDARG00000006031	fish	0.2479914	-0.0736010
ENSXETG00000013410	xenopus	0.3176923	-1.2910357
ENSDARG00000070447	fish	-0.0268868	-0.0312370
ENSXETG00000024933	xenopus	-0.3469115	1.4982524
ENSXETG00000020045	xenopus	-0.6369484	0.6133757

Table S5: Gene level measurements of codon optimality.

See the methods section "Measuring codon optimality at the gene level" and Additional file Figure S3. This table contains numerical measurements of codon optimality in some endogenous genes, we have generated two such measurements PLS1 and PLS2, positive values are associated with enrichment in optimal codons (stabilizing codon) and negative values with enrichment in non-optimal codons (destabilizing codon).

This table contains 57,627 rows and 4 columns.

Columns description:

- **gene_id ->**: ensembl gene id.
- **PLS1 ->** measurement 1 of codon optimality
- **PLS2 ->** measurement 2 of codon optimality
- **specie ->** vertebrate (human, mouse, xenopus, or zebrafish).

Table S5

gene_id	PLS1	PLS2	specie
ENSXETG00000027421	3.5829424	-1.9113006	xenopus
ENSMUSG00000031731	-1.0684200	2.9623095	mouse
ENSMUSG00000022000	-2.3651121	0.9490589	mouse
ENSG00000197647	-6.3191219	-5.4298493	human
ENSMUSG00000030538	0.4288678	-3.3887081	mouse

Table S6: Reporter sequences

This table contains 4 rows and 2 columns (Fig. 4e).

Columns description:

- **sequence_id ->**: The sequence id.
- **sequence ->** The reporter DNA sequence.

Next, you can find the first column of this table.

Table S6

sequence_id
CODING-optimal
CODING-non_optimal
3UTR-mir17
3UTR-mir17_mutant

Table S7: Codon frequencies of endogenous genes used to train machine learning model

This table contains the codon frequencies of the endogenous genes for zebrafish, *Xenopus*, mouse, and human. The frequencies were determined from the longest coding sequence isoform for each transcript.

Together this table and **Table S3** can be used to train the machine learning model to predict mRNA stability.

Columns description:

- **gene_id ->**: ensembl gene id.
- **AAA ->** frequency in transcript for codon AAA.
- **AAC ->** frequencies in transcript for codon AAC.
- etc.

Table S7

gene_id	AAA	AAC	AAG	AAT
ENSG00000175084	1	17	21	3
ENSMUSG00000005506	8	16	19	14
ENSMUSG00000053654	1	12	16	7
ENSG00000128928	3	7	21	13
ENSDARG00000025889	20	7	22	8
