# Supplementary material for: Crosstalk between codon optimality and cis-regulatory elements dictates mRNA stability
Source: Genome Biol. 2021 Jan 5;22:14. doi: 10.1186/s13059-020-02251-5 (PMC7783504; doi:10.1186/s13059-020-02251-5)
Supplement: Supplementary file 9 — Additional file 9:. Review history. [file 13059_2020_2251_MOESM9_ESM.docx]

Review History

**First round of review**

**Reviewer 1**

**Are you able to assess all statistics in the manuscript, including the appropriateness of statistical tests used?**

Yes, and I have assessed the statistics in my report.

**Comments to author:**

In the article, Medina et al. analyzed how codon composition and elements in the 3'UTR impact mRNA stability in embryonic development. The authors developed a machine learning model to predict mRNA stability. They demonstrate codon optimality as a key feature in maternal mRNA stability is both zebrafish and Xenopus. Furthermore, they show the interplay of codon optimality and other regulatory layers (microRNA and m6A) on mRNA stability. Overall, this work was well carried out, and sheds light on understanding the complexity of post-transcriptional control in embryonic development. However, the authors need to address a few issues to strengthen the work before the paper's acceptance.

Major:

1. The authors utilized a data set by Owens et al., which has samples in 30-minute intervals during Xenopus early embryogenesis. The authors chose 1 and 9 hpf to calculate mRNA stability. It would be helpful to get a sense of the robustness of these findings by using more time points.

2. This reviewer understands that codon optimality here was estimated in a holistic sense at the gene level. Since the regulatory effect of codons is translation-dependent, how does the relative position of optimal codons (and non-optimal codons) (ie, 5' end vs. 3'end) affect mRNA stability in this context of early embryogenesis?

3. The authors used imputation for missing data. But it is not clear how extensive the imputation is performed. Does the choice of imputation method influence the findings in any way?

Minor:

1. For Fig. 3a, the diagram is clean and easier to grasp, but it would also be useful to show the scatter plot and distribution of the actual data.

2. Fig 4a-d is quite busy as the authors probably surmised. Indicating the color for each quartile in the legend would be helpful for orientation as well.

3. There are many typos and grammatical issues (eg, Page 10: "all theses results evidence"; Page 13: some references to Fig 5 should be to Fig 6; Page 16: "regulatory" misspelled; Page 17: "researchers" misspelled; Fig. S4B: "enrichment" misspelled). Please proofread for other errors.

**Reviewer 2**

**Are you able to assess all statistics in the manuscript, including the appropriateness of statistical tests used?**

No, I do not feel adequately qualified to assess the statistics.

**Comments to author:**

The role of codon usage on the regulation of mRNA stability and translation have been shown in many organisms and cell types. In this manuscript the authors use computational modeling and transcriptome expression data to analyze the effect of codon optimality on maternal mRNA stability during the MZT of fish, frog, and mammals. The modeling data show that the effect of codon optimality is global and pervasive within the maternal transcriptome. Furthermore, the authors show that codon usage functions in an additive manner with other characterized mechanisms regulating maternal transcript degradation. In this way, the regulation of maternal transcript stability can be fine-tuned. These results provide insight into a widely acting layer of post-transcriptional regulation during the MZT. As mentioned in the manuscript, since this mechanism appears to be so wide-spread, understanding the impact of codon optimality may also provide information for how to exclude its effects on transcript stability to better isolate and understand the role of any other regulatory mechanisms to be studied in the future.

However, in this manuscript, the authors tend to over-complicate the interplay between codon-usage and other forms of regulation of mRNA stability, and they make several seemingly contradictory statements about how various mechanisms of post-transcriptional regulation may function in concert, either additively or antagonistically on the maternal transcriptome.

Major Points:

1) It is misleading and incorrect to make an absolute distinction between codon optimality functioning from the coding region, and all or most other regulatory mechanisms during the MZT functioning through regulatory elements in the 3'UTR. This was strongly implied throughout the manuscript as well as in the title. RNA-binding proteins do not exclusively bind to 3'UTRs. As examples: (1) the negative regulator of mRNA stability, Drosophila Smaug, binds mostly to ORFs (Chen et al. 2014); (2) the positive regulator of mRNA stability, G3BP/Rasputin shows enrichment of binding sites in ORFs (Laver et al. 2020); (3) the m6A RNA modification characterized in the zebrafish MZT is enriched around the start and stop codons and appears to be relatively depleted in the 3'UTR (Zhao et al., 2017). The authors should re-phrase these statements to reflect their intended distinction between protein-coding codon usage versus other features of mRNA (structure, primary sequence, modification).

2) Related to Point (1) above, in Figure 3E, the enrichment of m6A regulation in transcripts with negative residual scores might be higher if the entire transcript were accounted for, rather than just the 3'UTR. These data should be reanalyzed accordingly.

3) Figure 4E; page 10: The authors make a contradiction here. In the previous section (Page 9) they state the possibility that codon optimality likely functions additively with other regulatory mechanisms. All data presented here supports this hypothesis of additive regulation. There does not appear to be additional evidence that there is an "interaction" between codon optimality and miRNAs or m6A. Nor is the result presented in Figure 4E evidence that codon optimality functions as the "central stability determinant" (Page 10, Line 50), as both the effects of miRNAs and codon usage can be seen in Figure 3E, in addition to possibilities described below.

4) The authors further claim that "miRNAs dampen codon optimality effects" (Page 10, Line 57). This conclusion is an overstatement of the data presented in Figure 3E. Recent work uncovered the mechanism for mRNA degradation associated with non-optimal codons to the recruitment of the CCR4-NOT deadenylase complex by slow-moving ribosomes (Buschauer et al., 2020). Given that several other mechanisms of maternal transcript degradation also function through deadenylation, including miRNA/RISC, it is possible that degradation through deadenylation is at a maximum in non-optimal codon-containing reporter, such that miR-17 targeting cannot further downregulate reporter expression. This alternative hypothesis would account for comparable expression of the non-optimal reporter independent of miR-17 targeting. Thus, the authors should use additional reporters with sub-optimal or intermediate codon content to accurately assay the effect of codon optimality on the efficacy of miRNAs.

5) Figure 5B-C: (Note that it is not entirely clear what the y-axis represents so I may be confused.) Here, the authors seem to show that transcripts which have extremely non-optimal or extremely optimal codon content tend to be less regulated by miR-430-mediated repression. The authors do not address the possibility for subsets of transcripts that tend to be regulated by codon optimality, tending to have extremes in their codon usage, acting together with subsets of predominantly miRNA-regulated transcripts which tend to lack the need for extremes in codon usage. Here, it is implied that such a correlation shows direct interplay between the two mechanisms, and the authors conclude that miRNA targeting efficacy is directly affected by the coding sequence (seemingly excluding the possibility that the presence of one regulatory element may negatively correlate with the presence of the other on any given transcript). Note that the plots in Figure 6 seem to show that the vast majority of transcripts have a codon optimality level between -3 to 3, the range within which miRNA-mediated repression remains effective.

Minor Points:

1) The manuscript is inconsistent at denoting which datasets were used to produce specific figure panels; for example, Figure 2B displays data mRNA stability data from zebrafish and Xenopus embryos, and a citation is given for Bazzini et al, 2016. However, the referenced publication itself contains a set of stability data for both zebrafish and Xenopus, and it is therefore unclear whether the data for the zebrafish displayed in Figure 2B is taken from the newly acquired sequencing data or from the previously published zebrafish data.

2) In the methods section detailing the computational modeling, no information or references are given for the "64 codon frequencies" (page 21, 'Data allocation'). The measurement of these codon frequencies appears to be based on an analysis of the frequency of each codon in the transcriptome. It is unclear whether this input is newly computed or taken from the previously published dataset in Bazzini et al, 2016. Furthermore, it is unclear whether the authors take into account the effects of tRNA abundance and translation efficiency in their calculations.

3) The discussion pertaining to optimal and non-optimal codons defines these terms in relation to mRNA stability, with only a passing reference made to mRNA translation. However, other sources in the literature define codon optimality as "how efficiently the appropriate cognate tRNA can be selected from the cytoplasmic pool of tRNAs… [and] can be a powerful determinant of translation rates" (Hanson & Coller 2018). Furthermore, while correlations between codon content and codon optimality with mRNA stability are presented and discussed in the manuscript, no mechanism of action is proposed to explain how one affects the other (see Major Point 4 above). The manuscript would be strengthened by clarifying the language and terminology it uses to account for its use in the literature, and by a including discussion on the link between translation efficiency, codon bias, and mRNA stability and the mechanisms which link these phenomena.

4) Figure 4A-D: It is a hard to compare targets to non-targets of miR-430/-247, so it might be helpful to add a dotted line at y=0.

5) For Figures 5-6, please reorganise the panels to read in a logical order.

**Authors Response**

**Point-by-point responses to the reviewers’ comments:**

Reviewer 1

In the article, Medina et al. analyzed how codon composition and elements in the 3'UTR impact mRNA stability in embryonic development. The authors developed a machine learning model to predict mRNA stability. They demonstrate codon optimality as a key feature in maternal mRNA stability is both zebrafish and Xenopus. Furthermore, they show the interplay of codon optimality and other regulatory layers (microRNA and m6A) on mRNA stability. Overall, this work was well carried out, and sheds light on understanding the complexity of post-transcriptional control in embryonic development. However, the authors need to address a few issues to strengthen the work before the paper's acceptance.

Major 1: The authors utilized a data set by Owens et al., which has samples in 30-minute intervals during Xenopus early embryogenesis. The authors chose 1 and 9 hpf to calculate mRNA stability. It would be helpful to get a sense of the robustness of these findings by using more time points.

*We thank the reviewer for this comment because now, we can show the kinetics and robustness of our findings during the maternal to zygotic transition in Xenopus with a higher resolution.*

*To clarify what we have done: The machine learning model is still trained using the same Xenopus mRNA stability profile (Bazzini et al., 2016) because mRNA stability is calculated under transcription inhibition, alpha-amanitin treated embryos, to prevent zygotic expression (MZT), which is not present in Owens et al. In the previous version of the paper, we were using the log2 fold change of the mRNA level between 2- and 9-hours post-fertilization (hpf) to compare/validate the model during the MZT (active transcription) (Bazzini et al., 2016). First, we realized that in the text, we incorrectly wrote 1 hpf while in the figures, we correctly mentioned 2 hpf. We have corrected this error.*

*Now, following the reviewer’s suggestion, we incorporate the fold change between 2-and 5, 6, 7, 8, 9, and 10 hpf (new Figure S3d) to compare/validate the model (Owens et al., 2016). Moreover, we also reanalyzed the identification of cis-regulatory signals in the 3’ UTR with more time points (new Figure S5d) (Owens et al., 2016). We also incorporated more time points in the analysis showing the additive effect of miR-427 with codon optimality (new Figure S6a) (Owens et al., 2016). We also added multiple time points during zebrafish MZT (new Figures S3d, S5d and S6a). In summary, thanks to the reviewer’s comments, we have included more time points (Xenopus and zebrafish) in our analysis to support the robustness of the results.*

Major 2: This reviewer understands that codon optimality here was estimated in a holistic sense at the gene level. Since the regulatory effect of codons is translation-dependent, how does the relative position of optimal codons (and non-optimal codons) (ie, 5' end vs. 3'end) affect mRNA stability in this context of early embryogenesis?

*This is a very interesting point that we should have mentioned in the manuscript. It was previously suggested that the position of the codon along the transcript affects the codon-mediated regulation of mRNA stability in both yeast (Radhakrishnan et al., 2016) and zebrafish embryos (Mishima & Tomari, 2016).*

*In preliminary analyses, we have validated the effect of codon position (New Figure S2a). Our results indicated that non-optimal codons have a stronger destabilizing effect on the 3’ end than in the 5’ end of the coding sequence (New Figure S2a). We have also observed a similar trend in optimal codons (New Figure S2a). Optimal codons have a stronger stabilizing effect on the 3’ end than in the 5’ end. In summary, the effect of codon optimality is stronger in the 3’ end.*

*However, we did not observe a significant gain in predictive power by including codon positional information in the model (New Figure S2b). Indicating that the codon position has regulatory information, but globally the effect of the codon position is not a strong predictor. Therefore, we decided not to include it in our model.*

*However, we have updated the discussion to include this point, as it may be interesting to dissect the full grammar/rules of codon-mediated regulation in mRNA stability. For example, the order, and distribution of the codons may affect stability differently and might be use in the future to improve then mRNA stability prediction (New Figure S2c).*

Major 3: The authors used imputation for missing data. But it is not clear how extensive the imputation is performed. Does the choice of imputation method influence the findings in any way?

*We apologize for not clearly explaining the imputation strategy. We only used imputation for one variable: the length of the 3' UTR sequence. Only 5.4% of the genes were missing the 3' UTR sequence. The learning curve analysis suggests that if we exclude 5.4% of the genes, the predictive performance of the model is not affected (Figure S3b). Therefore, we opted to include these genes and use a conservative approach (mean) to impute the missing 3' UTR lengths. In sum, we have modified the methods section to describe the imputation strategy better.*

Minor 1: For Fig. 3a, the diagram is clean and easier to grasp, but it would also be useful to show the scatter plot and distribution of the actual data.

*While we included this figure as supplemental (Figure S5a), we did not reference it in the text. Now, we edited the manuscript to mention the figure and added the distribution to the scatter plot (Figure S5a).*

Minor 2: Fig 4a-d is quite busy as the authors probably surmised. Indicating the color for each quartile in the legend would be helpful for orientation as well.

*We appreciate the suggestion, and we have added the color information in the legend. Additionally, we added a horizontal line for easier comparison, as reviewer 2 suggested.*

Minor 3: There are many typos and grammatical issues (eg, Page 10: "all theses results evidence"; Page 13: some references to Fig 5 should be to Fig 6; Page 16: "regulatory" misspelled; Page 17: "researchers" misspelled; Fig. S4B: "enrichment" misspelled). Please proofread for other errors.

*We apologize for the typos and grammar errors and have sent the manuscript to our native English-speaking colleagues for proofreading. All the mentioned typos were corrected.*

Reviewer 2

The role of codon usage on the regulation of mRNA stability and translation have been shown in many organisms and cell types. In this manuscript the authors use computational modeling and transcriptome expression data to analyze the effect of codon optimality on maternal mRNA stability during the MZT of fish, frog, and mammals. The modeling data show that the effect of codon optimality is global and pervasive within the maternal transcriptome. Furthermore, the authors show that codon usage functions in an additive manner with other characterized mechanisms regulating maternal transcript degradation. In this way, the regulation of maternal transcript stability can be fine-tuned. These results provide insight into a widely acting layer of post-transcriptional regulation during the MZT. As mentioned in the manuscript, since this mechanism appears to be so wide-spread, understanding the impact of codon optimality may also provide information for how to exclude its effects on transcript stability to better isolate and understand the role of any other regulatory mechanisms to be studied in the future.

However, in this manuscript, the authors tend to over-complicate the interplay between codon-usage and other forms of regulation of mRNA stability, and they make several seemingly contradictory statements about how various mechanisms of post-transcriptional regulation may function in concert, either additively or antagonistically on the maternal transcriptome.

Major 1: It is misleading and incorrect to make an absolute distinction between codon optimality functioning from the coding region, and all or most other regulatory mechanisms during the MZT functioning through regulatory elements in the 3'UTR. This was strongly implied throughout the manuscript as well as in the title. RNA-binding proteins do not exclusively bind to 3'UTRs. As examples: (1) the negative regulator of mRNA stability, Drosophila Smaug, binds mostly to ORFs (Chen et al. 2014); (2) the positive regulator of mRNA stability, G3BP/Rasputin shows enrichment of binding sites in ORFs (Laver et al. 2020); (3) the m6A RNA modification characterized in the zebrafish MZT is enriched around the start and stop codons and appears to be relatively depleted in the 3'UTR (Zhao et al., 2017). The authors should re-phrase these statements to reflect their intended distinction between protein-coding codon usage versus other features of mRNA (structure, primary sequence, modification).

*We appreciate the reviewer's comment, and she/he is right, there are several examples of cis-regulatory elements in the coding sequences. We have rephrased the text, added the suggested references, and others (Chen et al., 2014; Hanson & Coller, 2018; Laver et al., 2020; Pinder & Smibert, 2013; Schoenberg & Maquat, 2012; Zhao et al., 2017), and we have modified the title. Moreover, we have included a new plot showing the effect of the m6A motif in the coding sequence (please see the next response).*

Major 2. Related to Point (1) above, in Figure 3E, the enrichment of m6A regulation in transcripts with negative residual scores might be higher if the entire transcript were accounted for, rather than just the 3'UTR. These data should be reanalyzed accordingly.

*In Figure 3D, we show that previously defined m6A targets in zebrafish (Zhao et al., 2017) display a negative residual score, indicating that m6A targets decay more than predicted by codon optimality. These genes contain enrichment of the m6A mark around the start and stop codons.*

*In contrast, the analysis presented in Figure 3e was looking specifically at the 3' UTR. In consideration of the reviewer's comment, we have done a new complimentary analysis separating maternal mRNAs based on the presence of the m6A motif (GGACT) in the coding or the 3'UTR in zebrafish and Xenopus (new Figure S5e). Supporting the reviewer comments, the maternal mRNAs with m6A motif in the coding region display lower residual score than the ones with m6A motif in the 3'UTR in both species. In sum, this result indicates that our model can also account for the repressive activity of cis elements (e.g. m6A) in the coding region.*

Major 3. Figure 4E; page 10: The authors make a contradiction here. In the previous section (Page 9) they state the possibility that codon optimality likely functions additively with other regulatory mechanisms. All data presented here supports this hypothesis of additive regulation. There does not appear to be additional evidence that there is an "interaction" between codon optimality and miRNAs or m6A. Nor is the result presented in Figure 4E evidence that codon optimality functions as the "central stability determinant" (Page 10, Line 50), as both the effects of miRNAs and codon usage can be seen in Figure 3E, in addition to possibilities described below.

*We thank the reviewer for pointing out this vague phrasing. We intended to communicate that both pathways can contribute toward regulating the level of expression; we did not mean to imply any type of "physical" interaction. To clarify this point, we have rephrased the statement. For the reporters used in Figure 4e, the influence of codon optimality is more potent than miR-17. For instance, the reporter enriched in optimal codons with a miR-17 target display a higher level of expression compared to the one enriched in non-optimal without a miR-17 target site. Therefore, in this particular case, we conclude that codon optimality is "stronger" than miR-17 in influencing transcript stability (of these reporters). However, as mentioned across the manuscript, it is the combination of all related pathways that will ultimately dictate the level of expression. For example, in the case of smarca2 mRNA that is enriched in optimal codons but contains three miR-430 seeds in the 3’ UTR (Laue, Rajshekar, Courtney, Lewis, & Goll, 2019), the microRNA repression is stronger and it is correctly destabilized during MZT. In this particular case (smarca2), miR-430 could be considered the central stability determinant.*

Major 4. The authors further claim that "miRNAs dampen codon optimality effects" (Page 10, Line 57). This conclusion is an overstatement of the data presented in Figure 3E. Recent work uncovered the mechanism for mRNA degradation associated with non-optimal codons to the recruitment of the CCR4-NOT deadenylase complex by slow-moving ribosomes (Buschauer et al., 2020). Given that several other mechanisms of maternal transcript degradation also function through deadenylation, including miRNA/RISC, it is possible that degradation through deadenylation is at a maximum in non-optimal codon-containing reporter, such that miR-17 targeting cannot further downregulate reporter expression. This alternative hypothesis would account for comparable expression of the non-optimal reporter independent of miR-17 targeting. Thus, the authors should use additional reporters with sub-optimal or intermediate codon content to accurately assay the effect of codon optimality on the efficacy of miRNAs.

*We appreciate the reviewer's comments, and we have rephrased the statement. Our results indicate that the microRNA target site in the 3'UTR can reduce the expression of mRNAs enriched in optimal codons. Particularly during the MZT, where miR-430/-427 is enriched in genes containing a high content of optimal codons but is nonetheless destabilized.*

*The idea that codon optimality and microRNA (or other regulatory pathways) compete or that their effect can become saturated in commonly used degradation pathways is an exciting idea which we have now incorporated into the discussion. While in yeast, it was shown that translation of non-optimal codons might trigger deadenylation via ribosomal binding of the CCR4-NOT subunit, NOT5, resulting in degradation (Buschauer et al., 2020), there are many differences in the vertebrate system. For example, while Dhh1p in yeast senses ribosome elongation speed and affects mRNA stability in a codon-dependent manner (Radhakrishnan et al., 2016), the role of the vertebrate ortholog, DDX6, in codon optimality has not been shown. We have analyzed DDX6 loss of function zebrafish embryos, and codon optimality was not affected.*

*While mRNAs enriched in optimal/non-optimal codons tend to contain longer/shorter poly(A)-tails in yeast (Radhakrishnan et al., 2016; Webster et al., 2018), zebrafish embryos (Bazzini et al., 2016; Mishima & Tomari, 2016) and human cells (Wu et al., 2019), this does not prove that optimality is dependent on poly(A) tail length. We have recently demonstrated that in both human cells and zebrafish embryos, reporters (optimal vs non-optimal), possessing either a poly(A)-tail or a histone tail (no poly(A)-tail) displayed similar trends of regulation based on codon composition (Wu et al., 2019). Therefore, the modulation in poly(A)-tail length is not required for codon-mediated changes in mRNA stability in human cells and zebrafish embryos, implying that poly(A)-tail shortening in non-optimal mRNAs likely occurs in parallel to or as a consequence of the destabilization. However, as we mentioned before, the idea that different pathways can compete for, or saturate, regulatory machinery is fascinating (now in discussion).*

*For the efficacy of the microRNA and additional reporters, please see the comments below.*

Major 5. Figure 5B-C: (Note that it is not entirely clear what the y-axis represents so I may be confused.) Here, the authors seem to show that transcripts which have extremely non-optimal or extremely optimal codon content tend to be less regulated by miR-430-mediated repression. The authors do not address the possibility for subsets of transcripts that tend to be regulated by codon optimality, tending to have extremes in their codon usage, acting together with subsets of predominantly miRNA-regulated transcripts which tend to lack the need for extremes in codon usage. Here, it is implied that such a correlation shows direct interplay between the two mechanisms, and the authors conclude that miRNA targeting efficacy is directly affected by the coding sequence (seemingly excluding the possibility that the presence of one regulatory

element may negatively correlate with the presence of the other on any given transcript). Note that the plots in Figure 6 seem to show that the vast majority of transcripts have a codon optimality level between -3 to 3, the range within which miRNA-mediated repression remains effective.

*We thank the reviewer for her/his comments. For clarification, the y-axis in previous Figure 5bc (now, Figure 5b and Figure S7a) represents a measure, estimated from the data, of the miR-430 repressive strength with respect to codon optimality (x-axis). For example, for an mRNA that is strongly repressed by miR-430, the miR-430 component will be larger (higher negative value in the y-axis). However, for another gene, where the miR-430 repression is weak, the miR-430 component is smaller (closer to 0 in the y-axis). Therefore, these results support the concept that the influence of miR-430 induced degradation is less potent in genes highly enriched in optimal or non-optimal codons.*

*The reviewer is undoubtedly correct in that most genes are of average optimality. Therefore, most of the genes containing miR-430 seed are destabilized, and for those, we can see an additive effect of both pathways (codon optimality and miR-430, Figure 4). However, in the more extreme codon optimality scores, the miR-430 component appears to be diluted, but as the reviewer mentioned, the number of mRNAs in the extreme situation is limited.*

*Regarding the possibility that the "presence of one regulatory element may negatively correlate with the presence of the other on any given transcript," it is essential to mention that in this analysis (Figure 5a), we only analyzed mRNAs that contain a miR-430 seed site. Hence, all of the transcripts in this analysis include the cis element (miR-430 target site), but not all are equally functional (Figure 5a), dicer vs. wild-type, and Figure 5b, prediction of miR-430 activity). While we cannot rule out that other cis-regulatory elements might be affecting the results, our reporters (Figure 4e), contain almost identical sequences (optimal and non-optimal differing by only a single nucleotide insertion creating a frameshift). Therefore, it is unlikely that one of these reporters has other elements affecting stability while other does not.*

*Based on the comment "the authors should use additional reporters with sub-optimal or intermediate codon content to accurately assay the effect of codon optimality on the efficacy of miRNAs," we have analyzed a massive reporter library (Bazzini et al., 2016) in zebrafish and Xenopus, providing further evidence that microRNA efficacy can be affected by codon optimality.*

*This massive reporter library allows us to use thousands (zebrafish: 2 hrs = 61,981 and 8 hrs = 61,388; Xenopus 1 hr = 23,927 and 9 hrs = 52,016) of injected mRNAs into zebrafish and Xenopus embryos with random coding and 3’UTR sequences to address the efficacy of miR-430/-427 (new Figure 5c-e, new Figure S7b-e).*

*We analyzed the codon content and depletion miR-430/-427 targets in the massive library before and after the MZT, and observed that the miR-430/-427 seed depletion is consistently more robust in mRNAs with neutral or average codon optimality in zebrafish (New Figure 5d) and Xenopus embryos (New Figure 5e). Consistent with the endogenous genes (Figure 5ab and Figure S7A) and individual reporters (Figure 4e), these results provide additional information supporting the hypothesis that microRNA targeting efficacy can be affected by coding sequences highly enriched in optimal codons or non-optimal codons.*

Minor 1. The manuscript is inconsistent at denoting which datasets were used to produce specific figure panels; for example, Figure 2B displays data mRNA stability data from zebrafish and Xenopus embryos, and a citation is given for Bazzini et al, 2016. However, the referenced publication itself contains a set of stability data for both zebrafish and Xenopus, and it is therefore unclear whether the data for the zebrafish displayed in Figure 2B is taken from the newly acquired sequencing data or from the previously published zebrafish data.

*We have now included references specifying which data sets were used for each analysis.*

Minor 2. In the methods section detailing the computational modeling, no information or references are given for the "64 codon frequencies" (page 21, 'Data allocation'). The measurement of these codon frequencies appears to be based on an analysis of the frequency of each codon in the transcriptome. It is unclear whether this input is newly computed or taken from the previously published dataset in Bazzini et al, 2016. Furthermore, it is unclear whether the authors take into account the effects of tRNA abundance and translation efficiency in their calculations.

*All of the codon frequencies were calculated based on the frequency of each codon in the transcriptome. For each gene, we took the longest isoform to calculate the codon frequencies. Now, we include a new Table S7 with the codon frequency of all the transcripts used in this study (from zebrafish, Xenopus, mouse, and human). We thank the reviewer for this comment because addressing it will better allow other labs to use our data.*

*We have not taken into account the level of tRNA nor translation efficiency in this analysis. While we previously demonstrated a positive correlation between codon optimality and tRNA abundance in zebrafish embryos (Bazzini et al., 2016) and in human cells (Wu et al., 2019), tRNA quantifications are still not very robust. Though upon analysis of two different tRNA profiles (Evans, Clark, Zheng, & Pan, 2017; Gogakos et al., 2017) we observed significant correlation with codon optimality, however, the tRNA profiles did not display a robust correlation to each other (Wu et al., 2019). Moreover, we have shown that the ratio of charged tRNA ("ready-to-go" with the amino acid) compared to the total (charged plus uncharged) also correlates with codon optimality, indicating that not only the level but the quality of the tRNA might dictate the codon optimality effect on mRNA stability (Wu et al., 2019). Additionally, we are still missing the element of tRNA modifications in any of these analyses. Therefore, while this is certainly an excellent idea, due to these limitations, we are still not able to incorporate tRNA into the model.*

*We have also published data on the effect of codon optimality on translation efficiency (Wu et al., 2019). However, as translation efficiency can change (Bazzini, Lee, & Giraldez, 2012), it would be very challenging and potentially misleading to incorporate it into our model. We have, however, included the idea that cis elements can also affect the level of translation (likely translation initiation through the 5' and 3' UTRs) and, consequently, affect the effect of codon optimality on mRNA stability.*

3) The discussion pertaining to optimal and non-optimal codons defines these terms in relation to mRNA stability, with only a passing reference made to mRNA translation. However, other sources in the literature define codon optimality as "how efficiently the appropriate cognate tRNA can be selected from the cytoplasmic pool of tRNAs... [and] can be a powerful determinant of translation rates" (Hanson & Coller 2018). Furthermore, while correlations between codon content and codon optimality with mRNA stability are presented and discussed in the manuscript, no mechanism of action is proposed to explain how one affects the other (see Major Point 4 above). The manuscript would be strengthened by clarifying the language and terminology it uses to account for its use in the literature, and by a including discussion on the link between translation efficiency, codon bias, and mRNA stability and the mechanisms which link these phenomena.

*We have used the words optimal and non-optimal as the ability of a particular codon to affect the stability of an mRNA in cis. As described in Major point 4, there are several differences between codon optimality in vertebrates and yeast, and the mechanism is not entirely clear. For example, it has been proposed that deadenylation was part of the mechanism due to the strong correlation between codon content and poly(A) tail length (yeast, zebrafish, human). However, we have shown that reporters with histone tails (no poly(A) tail) are also regulated by the codon optimality mechanism, demonstrating that the poly(A) tail is not required (Wu et al., 2019). Following our previous comments (Major point 4), we have modified the discussion.*

4)	Figure 4A-D: It is a hard to compare targets to non-targets of miR-430/-247, so it might be helpful to add a dotted line at y=0.

*Thank you, we have added a line.*

5)	For Figures 5-6, please reorganize the panels to read in a logical order. Thank you, we have reorganized the panels.

*For both reviewers:*

*During the uploading of the raw data, we identified that there were few mutations in the 3’UTR used in original figure 4e and S4e. While it was unlikely that those mutations would affect the results, we repeated the cloning to be 100% sure that the only differences between the 3’UTR with miR-17 target site and its counterpart (Mut) were two mutations in the target site. As expected, the results were similar. We want to thank the editors because in our Covid-19 situation, it took us a month to do this simple experiment.*

*Bazzini, A. A., Lee, M. T., & Giraldez, A. J. (2012). Ribosome profiling shows that miR-430 reduces translation before causing mRNA decay in zebrafish. Science, 336(6078), 233¬237.*

*Buschauer, R., Matsuo, Y., Sugiyama, T., Chen, Y.-H., Alhusaini, N., Sweet, T., . . . Nobuta, R. (2020). The Ccr4-Not complex monitors the translating ribosome for codon optimality. Science, 368(6488).*

*Chen, L., Dumelie, J. G., Li, X., Cheng, M. H., Yang, Z., Laver, J. D., . . . Lipshitz, H. D. (2014). Global regulation of mRNA translation and stability in the early Drosophila embryo by the Smaug RNA-binding protein. Genome biology, 15(1), 1-21.*

*Evans, M. E., Clark, W. C., Zheng, G., & Pan, T. (2017). Determination of tRNA aminoacylation*

*levels by high-throughput sequencing. Nucleic acids research, 45(14), e133-e133.*

*Gogakos, T., Brown, M., Garzia, A., Meyer, C., Hafner, M., & Tuschl, T. (2017). Characterizing expression and processing of precursor and mature human tRNAs by hydro-tRNAseq and PAR-CLIP. Cell reports, 20(6), 1463-1475.*

*Hanson, G., & Coller, J. (2018). Codon optimality, bias and usage in translation and mRNA decay. Nature reviews Molecular cell biology, 19(1), 20-30.*

*Laue, K., Rajshekar, S., Courtney, A. J., Lewis, Z. A., & Goll, M. G. (2019). The maternal to zygotic transition regulates genome-wide heterochromatin establishment in the zebrafish embryo. Nature communications, 10(1), 1-10.*

*Laver, J. D., Ly, J., Winn, A. K., Karaiskakis, A., Lin, S., Nie, K., . . . Khademi, A. (2020). The RNA-Binding Protein Rasputin/G3BP Enhances the Stability and Translation of Its Target mRNAs. Cell reports, 30(10), 3353-3367. e3357.*

*Mishima, Y., & Tomari, Y. (2016). Codon usage and 3ʹ UTR length determine maternal mRNA stability in zebrafish. Molecular cell, 61(6), 874-885.*

*Owens, N. D., Blitz, I. L., Lane, M. A., Patrushev, I., Overton, J. D., Gilchrist, M. J., . . . Khokha, M. K. (2016). Measuring absolute RNA copy numbers at high temporal resolution reveals transcriptome kinetics in development. Cell reports, 14(3), 632-647.*

*Pinder, B. D., & Smibert, C. A. (2013). Smaug: an unexpected journey into the mechanisms of post-transcriptional regulation. Fly, 7(3), 142-145.*

*Radhakrishnan, A., Chen, Y.-H., Martin, S., Alhusaini, N., Green, R., & Coller, J. (2016). The DEAD-box protein Dhh1p couples mRNA decay and translation by monitoring codon optimality. cell, 167(1), 122-132. e129.*

*Schoenberg, D. R., & Maquat, L. E. (2012). Regulation of cytoplasmic mRNA decay. Nature Reviews Genetics, 13(4), 246-259.*

*Webster, M. W., Chen, Y.-H., Stowell, J. A., Alhusaini, N., Sweet, T., Graveley, B. R., . . . Passmore, L. A. (2018). mRNA deadenylation is coupled to translation rates by the differential activities of Ccr4-Not nucleases. Molecular cell, 70(6), 1089-1100. e1088.*

*Wu, Q., Medina, S. G., Kushawah, G., DeVore, M. L., Castellano, L. A., Hand, J. M., . . . Bazzini, A. A. (2019). Translation affects mRNA stability in a codon-dependent manner in human cells. Elife, 8, e45396.*

*Zhao, B. S., Wang, X., Beadell, A. V., Lu, Z., Shi, H., Kuuspalu, A., . . . He, C. (2017). m 6 A-dependent maternal mRNA clearance facilitates zebrafish maternal-to-zygotic transition. Nature, 542(7642), 475-478.*

**Second round of review**

**Reviewer 2**

The authors have done an excellent job of revising their manuscript and responding to my concerns. It is much improved.
